# Targeting MTHFD2 alters metabolic homeostasis and synergizes with bortezomib to inhibit multiple myeloma

**DOI:** 10.1038/s41420-025-02498-6

**Published:** 2025-04-25

**Authors:** Mingyuan Jia, Ze Fu, Chenjing Ye, Wenbin Xu, Jia Liu, Chengyu Wu, Hua Yan

**Affiliations:** 1 Shanghai Institute of Hematology, State Key Laboratory of Medical Genomics, National Research Center for Translational Medicine at Shanghai, Ruijin Hospital, Shanghai Jiao Tong University School of Medicine, Shanghai, China; 2 Department of General Practice, Ruijin Hospital, Shanghai Jiao Tong University School of Medicine, Shanghai, China

**Keywords:** Myeloma, Cancer metabolism

## Abstract

Multiple myeloma (MM) is an incurable hematologic malignancy. While recent therapies have significantly improved survival in MM patients, drug resistance and refractory phenomenon underscores the urgent need of new therapeutic targets. Methylenetetrahydrofolate dehydrogenase 2(MTHFD2) has been widely reported as a potential and promising anti-cancer target, but its role and underlying mechanisms remain unclear in MM. We aimed to investigate the biologic function and mechanisms of MTHFD2 in MM. First, we demonstrated that MTHFD2 is overexpressed in MM and associated with poor prognosis. We then illustrated that targeting MTHFD2 exhibits anti-MM effects in vitro and in vivo. Mechanistically, targeting MTHFD2 inhibited glycolysis and mitochondrial respiration in MM cells. For the nonmetabolic function of MTHFD2, we found that MTHFD2 knockdown affected the unfolded protein response (UPR) via decreasing expression of the splice form of X-box binding protein 1 (XBP1s). Importantly, the level of MTHFD2 in MM cells was associated with sensitivity of bortezomib, and targeting MTHFD2 synergizes with bortezomib against MM in vitro and in vivo. In summary, our innovative findings suggest that MTHFD2 is a promising target for MM, targeting it alters metabolic homeostasis of MM and synergizes with bortezomib to inhibit MM.

## Introduction

Multiple myeloma (MM) is a hematologic malignancy resulting from malignant proliferation of abnormal plasma cells in the bone marrow, which accounts for 10% of all hematologic malignancies [1, 2]. Abnormal plasma cells in MM often secret monoclonal immunoglobulin protein, resulting in MM mostly in a chronic endoplasmic reticulum (ER) stress state and requiring the unfolded protein response (UPR) to regulate this stress state [3]. The pathogenesis of MM is complex, and studies have reported the presence of metabolic disorders in MM, including abnormal lipid metabolism, glucose metabolism, nucleotide metabolism, and amino acid metabolism, which play an important role in the development of MM [4–8]. Currently, therapies for MM mainly include proteasome inhibitors (PIs) (such as bortezomib and carfilzomib), immunomodulators (such as lenalidomide and pomalidomide), and immune-related therapies [such as CD38 and B cell maturation antigen (BCMA) monoclonal antibodies, bispecific monoclonal antibodies], and chimeric antigen receptor T cell (CAR-T) therapy [9–14]. These therapies have significantly improved the prognosis of MM patients, however, relapse and refractory phenomenon of MM remains common [2].

One carbon (1C) metabolism plays an important role in amino acid homeostasis, purine and pyrimidine synthesis, epigenetic maintenance and oxidative stress in cells [15]. Methylenetetrahydrofolate dehydrogenase 2 (MTHFD2) is a bifunctional enzyme with dehydrogenase and cyclohydrolase activities in cellular mitochondria, which acts as a key catalytic enzyme for 1C metabolism [16]. During embryonic development, MTHFD2 supports rapid proliferation of cells through enzymatic reactions, and MTHFD2 is lowly or not expressed in most adult tissues, whereas in tumors, MTHFD2 is reactivated and highly expressed in a variety of cancers [17–19]. It has previously been reported that MTHFD2 plays an important role in various tumor types such as gastric cancer, colon cancer, renal cancer, acute myeloid leukemia (AML), lung cancer, bladder cancer, and prostate cancer. In terms of mechanism, MTHFD2 plays an important role in the malignant biological behavior of various tumors mainly by regulating oxidative stress, RNA N6-methyladenosine (m6A) modification, DNA damage repair, glycolysis, mitochondrial function, and nonmetabolic function [20–26]. However, the biological functions and mechanisms of MTHFD2 in MM remain elusive.

In response to PIs, which predominantly inhibit the proteasome 26S subunit, thereby aggravating ER stress, and then decompensated ER stress conditions trigger terminal UPR, ultimately leading to cell death [27, 28]. According to current reports, PI treatment decreases mitochondrial respiratory capacity, which leads to metabolic dysfunction in MM cells [29]. PI-resistant MM cell lines have increased activity of UPR [30], which consists of three pathways, inositol-requiring enzyme 1α (IRE1α)/X-box binding protein 1 (XBP1), PKR-like ER kinase (PERK), and activating transcription factor 6 (ATF6) [31]. In the UPR, IRE1α activates its endoribonuclease following oligomerization and autophosphorylation, thereby splicing XBP1 mRNA to remove its 26-nucleotide intron, eventually XBP1 becomes the splice form of X-box binding protein 1 (XBP1s) [32]. The IRE1α-XBP1 pathway plays a pro-survival role in the UPR [33]. It has been reported that dysfunctional XBP1s plays an important role in the pathogenesis of MM, and targeting the IREa/XBP1s pathway is an important means for the treatment of MM [34, 35].

In this study, we investigated the main biological role of MTHFD2 in MM. We found that MTHFD2 is highly expressed in MM patients and is associated with poor prognosis. Subsequently, we found that targeting MTHFD2 could significantly inhibit MM proliferation and promote apoptosis in vitro and in vivo. Mechanistically, we found that targeting MTHFD2 significantly inhibited glycolysis and oxidative phosphorylation in MM cells, and MTHFD2 knockdown decreased overall cellular SAM levels and RNA m6A modification levels. Furthermore, we analyzed public database (GSE24080) and found that MTHFD2 mainly affected the UPR response. The knockdown of MTHFD2 significantly down-regulated the expression of XBP1s via nonmetabolic function. Based on the above results, we hypothesized that MTHFD2 may be associated with drug sensitivity to PIs, and subsequent assays demonstrated that targeting MTHFD2 has a significant synergistic anti-MM effect with bortezomib in vivo and in vitro. In conclusion, MTHFD2 is a novel target for MM therapy and targeting MTHFD2 synergizes with bortezomib against MM, thus providing new ideas for clinical MM treatment options.

## Results

### MTHFD2 is overexpressed in MM and associated with poor prognosis

**Fig. 1:**
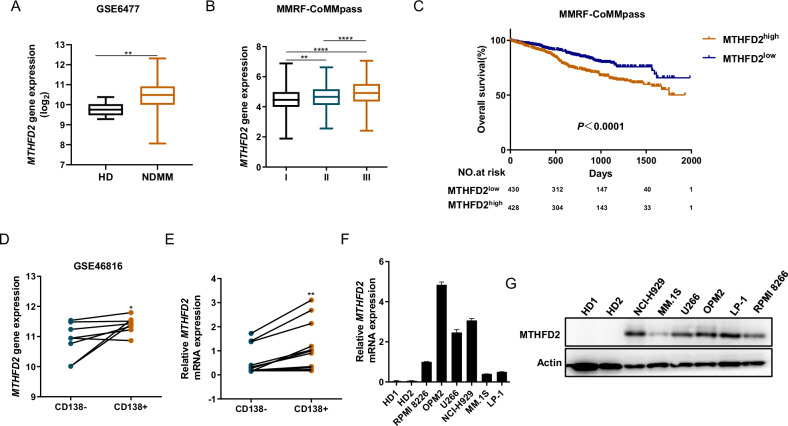
MTHFD2 is overexpressed in MM and associated with poor prognosis. **A** The MTHFD2 expression data from GSE6477 were analyzed in healthy donors (HD, *n* = 15) and newly diagnosed MM (NDMM, *n* = 69). **B** The MTHFD2 expression data of MM patients with different ISS stage from MMRF-CoMMpass dataset, ISS I stage (*n* = 281), ISS II stage (*n* = 298), ISS III stage (*n* = 258). **C** In the MMRF-CoMMpass dataset, Kaplan–Meier curves showed that overall survival of the patients with MTHFD2 high expression (MTHFD2^high^, *n* = 428) and MTHFD2 low expression (MTHFD2^low^, *n* = 430). **D** Comparisons of MTHFD2 expression in GSE46816 were shown, including eight pairs of CD138-positive (CD138+) and CD138-negative (CD138-) MM cells. **E** Relative MTHFD2 expression between MACS-sorted CD138+ and CD138- BMMCs from MM patients was detected using qRT-PCR normalized to ACTIN. **F** QRT-PCR assay showed that relative MTHFD2 expression normalized to ACTIN in PBMCs from healthy donor 1(HD1), PBMCs from healthy donor 2 (HD2), and MM cell lines including NCI-H929, OPM2, U266, LP-1, MM.1S, RPMI 8266. **G** Western blot assay was used to detect the MTHFD2 and Actin protein level of PBMCs from healthy donor 1 (HD1),PBMCs from healthy donor 2 (HD2), and MM cell lines including NCI-H929, OPM2, U266, LP-1, MM.1S, RPMI 8266. The data are presented as mea*n* ± SD. Student’s *t* test was used to compare the differences between two groups. (*P < 0.05, **P < 0.01, ****P < 0.0001).

MTHFD2 acts as an important enzyme in 1C metabolism, which provides important metabolites for cell survival and proliferation, especially rapidly proliferating tumor cells [17]. To investigate its role in MM, we analyzed data from GSE6477 dataset and found that the expression level of MTHFD2 was higher in newly diagnosed MM patients (NDMM) compared with healthy donors (HD) (Fig. 1A). Interestingly, the expression level of MTHFD2 is not only increased with the ISS stage of MM patients but also associated with poor overall survival (OS) in MMRF-CoMMpass dataset (Fig. 1B, C). In GSE46816, the expression level was higher in CD138+ cells which represented MM character than CD138- cells (Fig. 1D). Consistent with this, we confirmed that the level of MTHFD2 was higher in CD138+ cells sorted from BMMCs of MM patients compared CD138- cells (Fig. 1E). Then we analyzed the mRNA and protein levels of MTHFD2 across healthy donors and various MM cell lines, and found that the level of MTHFD2 was significant higher compared with HD and widely expressed in MM cell lines (Fig. 1F, G). Overall, the expression of MTHFD2 was increased with MM progression and associated with poor OS. According to the above data, we hypothesized that MTHFD2 may play an important role in MM.

### MTHFD2 knockdown inhibits proliferation, induces apoptosis and causes G0/G1 arrest in MM cells

**Fig. 2:**
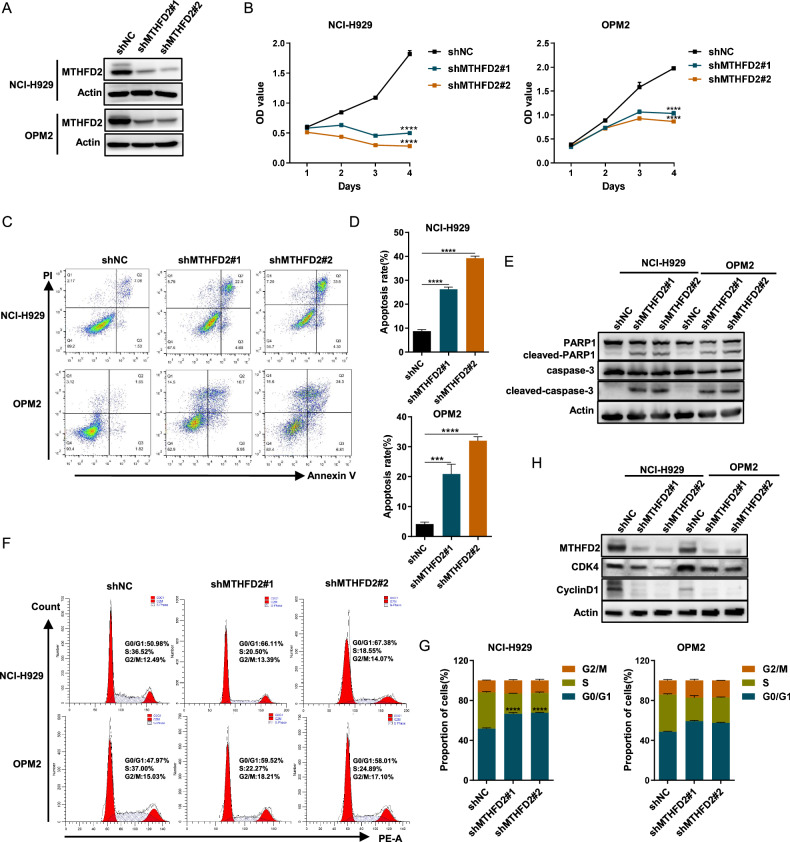
MTHFD2 knockdown inhibits proliferation, induces apoptosis and causes G0/G1 arrest in vitro. **A** Western blot assay showing the MTHFD2 and actin protein level of shNC (Negative control), shMTHFD2#1, shMTHFD2#2 in NCI-H929 and OPM2 cells. **B** CCK8 assay was used to quantitatively detect the proliferation of MTHFD2 knockdown NCI-H929 and OPM2 cells using OD values at the corresponding time. Data were presented as mean ± SD from three independent experiments. **C** Annexin V /PI double staining flow cytometry was used to detect the apoptotic rate after knockdown of MTHFD2 in NCI-H929 and OPM2 cells. **D** Results as bar graph are calculated percentages of Annexin-V positive cell populations from three independent experiments. **E** The apoptosis related proteins including PARP1, cleaved-PARP1, caspase3, cleaved-caspase3, and internal reference actin were detected by western blot assay in MTHFD2 knockdown NCI-H929 and OPM2 cells. **F** Cell cycle of NCI-H929 and OPM2 cells was examined after knockdown of MTHFD2.Results were presented as peak plots using Modfit. **G** The distributions of cells in G0/G1, S and G2/M phases were calculated. Data represent mean ± SD from triplicate independent experiments. **H** Western blot showed protein expression of cyclin D1 and CDK4 in MTHFD2-silenced NCI-H929 and OPM2 cells. The data are presented as mea*n* ± SD. Student’s *t* test was used to compare the differences between two groups. (***P < 0.001, ****P < 0.0001).

To explore the potential functions of MTHFD2 in MM, we performed loss-of-function experiments using NCI-H929 and OPM2 cells with relatively higher levels of MTHFD2. First, we found that MTHFD2 knockdown significantly inhibited proliferation in NCI-H929 and OPM2 cells, and western blot assays confirmed knockdown efficiency (Fig. 2A, B). Next, we detected apoptosis by Annexin V/PI double staining following MTHFD2 knockdown in NCI-H929 and OPM2 cells. We found that MTHFD2 knockdown induced apoptosis in MM cells, concomitant with an increase in cleaved-PARP 1 and cleaved-caspase3 (Fig. 2C–E). Flow cytometry illustrated that knockdown of MTHFD2 caused G0/G1 arrest, which was accompanied by a decrease in G0/G1-related CDK4 and cyclinD1 (Fig. 2F–H). In conclusion, the above findings suggest that MTHFD2 plays a critical role in MM malignancy. MTHFD2 knockdown inhibits MM in vitro.

### MTHFD2 knockdown inhibits MM malignancy in vivo

**Fig. 3:**
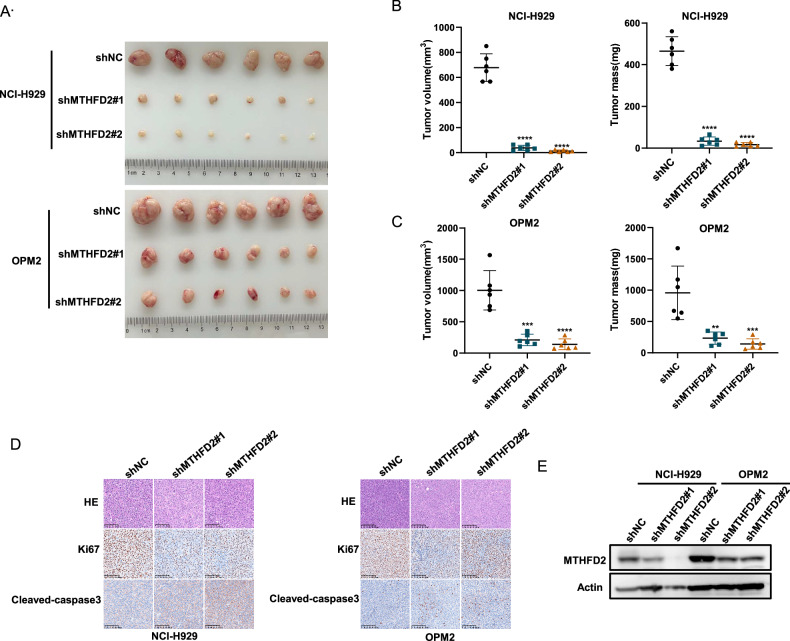
MTHFD2 knockdown inhibits MM malignancy in vivo. **A** NCI-H929 and OPM2 cells stably infected with shNC, shMTHFD2#1 or shMTHFD2#2 lentivirus were subcutaneously injected into NOG mice to establish MM xenograft mouse models (*n* = 6 per group). Tumors from mice described above were dissected and photographed on day 21. **B**, **C** Tumor volume and weight were then assessed. These mouse experiments were repeated once. **D** Paraffin-embedded tumor sections of shNC, shMTHFD2#1 and shMTHFD2#2 groups were stained with hematoxylin and eosin (HE), Ki67 and cleaved caspase-3 antibodies (scale bar 100 μm). **E** Western blot assay was used to confirm the MTHFD2 knockdown efficiency of tumors from shNC, shMTHFD2#1 and shMTHFD2#2 groups. The data are presented as mean ± SD. Student’s *t* test was used to compare the differences between two groups (**P < 0.01, ***P < 0.001, ****P < 0.0001).

Based on the above findings, we further explored the effect of MTHFD2 knockdown in vivo. We used MTHFD2-knockdown NCI-H929 and OPM2 cells to construct MM mouse xenograft subcutaneous tumor model. As shown in Fig. 3A, tumors were smaller in the shMTHFD2 group compared with shNC group, reflecting by significant reduction in tumor volume and weight (Fig. 3B, C). Otherwise, immunohistochemistry of tumors showed significantly lower level of Ki67 and higher level of cleaved-caspase 3 in the shMTHFD2 group (Fig. 3D). The knockdown efficiency of MTHFD2 in tumors was confirmed by western blot assay (Fig. 3E). These data suggest that MTHFD2 knockdown inhibits MM proliferation and promotes apoptosis in vivo.

### DS18561882, the inhibitor targeting MTHFD2, exhibits anti-MM effects in vitro

**Fig. 4:**
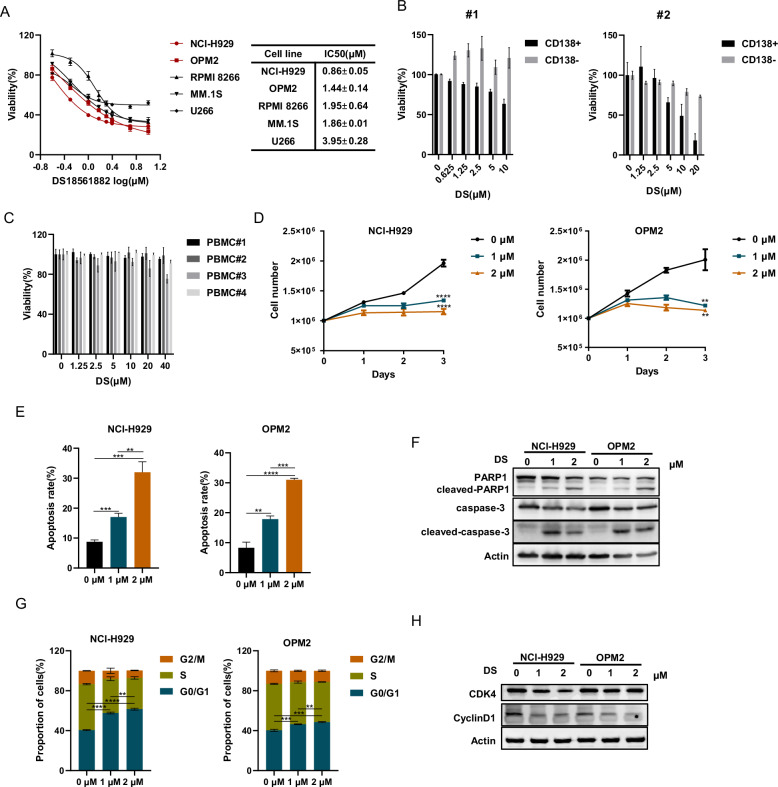
DS18561882, the inhibitor targeting MTHFD2, exhibits anti-MM effects in vitro. **A** CCK-8 assay was used to examine the effect of DS18561882 (DS) on MM cells (NCI-H929, OPM2, U266, MM.1S, RPMI 8266) at the indicated concentrations for 48 h. IC50 was calculated from the dose concentration response curve and presented as mean ± SD from three independent experiments. **B** The CD138+ and CD138− cells sorted from BMMCs of MM patients (#1, #2) were added DS at indicated concentrations for 48 h and the cell viability was examined by CCK-8 assay. **C** PBMCs from healthy donors were tested for cell viability after 48 h of DS treatment at the indicated concentrations. **D** The cell number was counted at indicated time with the treatment of DS (0, 1 and 2 μM) in NCI-H929 and OPM2 cells. Data represent mean ± SD from triplicate independent experiments. Student ‘s *t* test was used for two group analysis on day 3. **E** The apoptosis rate of NCI-H929 and OPM2 cells with DS (0, 1 and 2 μM) for 48 h was examined by flow cytometry, then results as bar graph were calculated percentages of Annexin-V positive cell populations from three independent experiments. **F** The apoptosis related proteins after treatment of DS (0, 1 and 2 μM) including PARP1, cleaved-PARP1, caspase3 and cleaved-caspase3 were tested by western blot assay. **G** Cell cycle was detected by Flow cytometry in NCI-H929 and OPM2 cells with DS (0, 1 and 2 μM) for 48 h. Data represent mean ± SD from triplicate independent experiments. **H** Western blot showed protein expression of cyclin D1 and CDK4 in NCI-H929 and OPM2 cells with DS (0, 1 and 2 μM) for 48 h. The data are presented as mean ± SD. Student’s *t* test was used to compare the differences between two groups. (**P < 0.01, ***P < 0.001, ****P < 0.0001).

DS18561882 (DS) was a specific inhibitor targeting MTHFD2 [36]. We used this inhibitor to further explore the effect of MTHFD2 on MM in order to provide more theoretical basis for clinical trials of this drug, so as to achieve clinical translation for the treatment of MM. First, we treated various MM cell lines with DS at the indicated concentration for 48 h. The half maximal inhibitory concentration (IC50) was calculated by dose–response curves to clarify the effect of DS on MM cells. As shown in Fig. 4A, we confirmed that DS inhibited the proliferation of various MM cell lines with IC50 ranging from 0.86 ± 0.05 μM to 3.95 ± 0.28 μM. Then we illustrated that DS showed concentration-dependent inhibition of proliferation of CD138+ cells, but a weak inhibitory effect on CD138- cells, both sorted from BMMCs of MM patients (Fig. 4B). This data suggested that DS showed some selectivity for CD138+ cells. To further investigate the toxic effects of DS, we treated PBMCs of healthy donors with DS and found that DS showed little toxicity to PBMCs even at a maximum concentration of 40 μM (Fig. 4C). We subsequently performed experiments using NCI-H929 and OPM2, which were relatively sensitive to DS, and found that DS could significantly inhibited cell proliferation, induced apoptosis and caused G0/G1 arrest at the indicated concentrations, leading to an increase in apoptosis related proteins and a decrease in G0/G1 related proteins (Figs. 4D–H and S1A, B). Overall, these results indicate that DS exhibits anti-MM effects in vitro.

### DS18561882 exhibits anti-MM effects in vivo

**Fig. 5:**
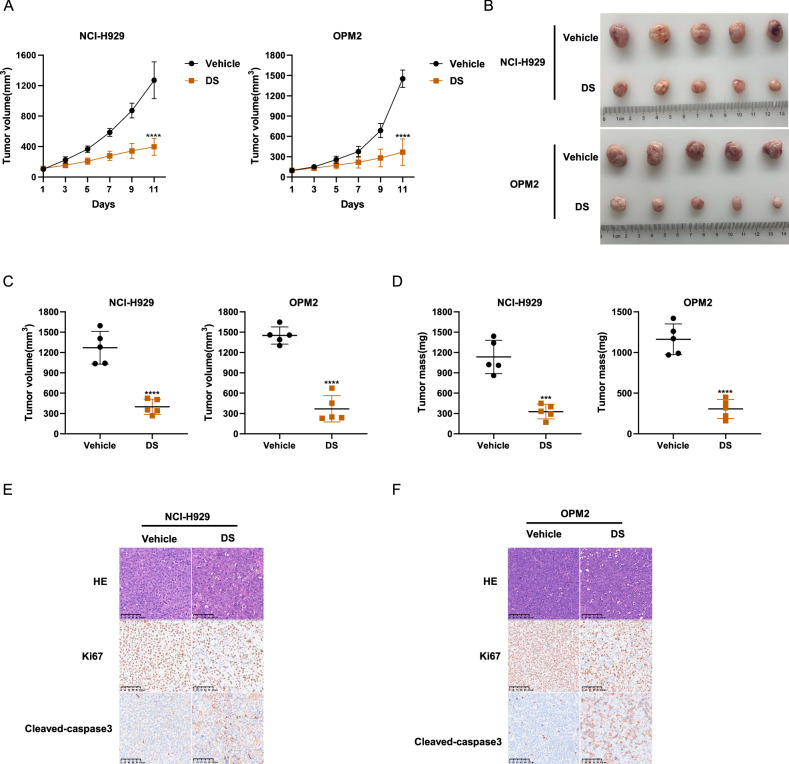
DS18561882 exhibits anti-MM effects in vivo. **A** NCI-H929 and OPM2 cells were subcutaneously injected into NOG mice to establish MM xenograft mouse models. Mice of the two cell lines were randomly divided into two groups (vehicle and DS group), respectively (*n* = 5 per group). Tumor volumes represented by line graphs were recorded every other day after treatment (DS 150 mg/kg/day). **B** Tumors from these mice were dissected and photographed 11 days after administration of vehicle or DS (150 mg/kg, oral gavage). **C**, **D** The Tumor volume and weight were then assessed. These mouse experiments were repeated once. **E**, **F** Paraffin-embedded tumor sections of vehicle and DS group were stained with hematoxylin and eosin (HE), Ki67 and cleaved caspase-3 antibodies (scale bar 100 μm). The data are presented as mean ± SD. Student’s *t* test was used to compare the differences between two groups. (***P < 0.001, ****P < 0.0001).

To further explore the effect on MM of DS in vivo, we used NCI-H929 and OPM2 cells to construct MM mouse xenograft subcutaneous tumor model. When xenograft tumors of mice were measurable, we randomized mice into vehicle and DS groups. Then two groups of mice were administered vehicle or DS (150 mg/kg/day) for 11 days. As shown in Fig. 5A, the tumor proliferation rate in DS group was significantly lower than that in vehicle group, and no statistically significant weight loss was observed in mice of DS group (Fig. S2A). In the end of this experiment, the tumors in DS group were significantly smaller than those in vehicle group, reflecting by significant reduction in tumor volume and weight (Fig. 5B–D). Consistent with the above data, immunohistochemistry of these tumors in the DS group showed lower Ki67 and significantly increased cleaved-caspase3 (Fig. 5E, F). These data suggest that DS exhibits anti-MM effects in vivo.

### MTHFD2 knockdown leads to metabolic dysfunction in MM cells

**Fig. 6:**
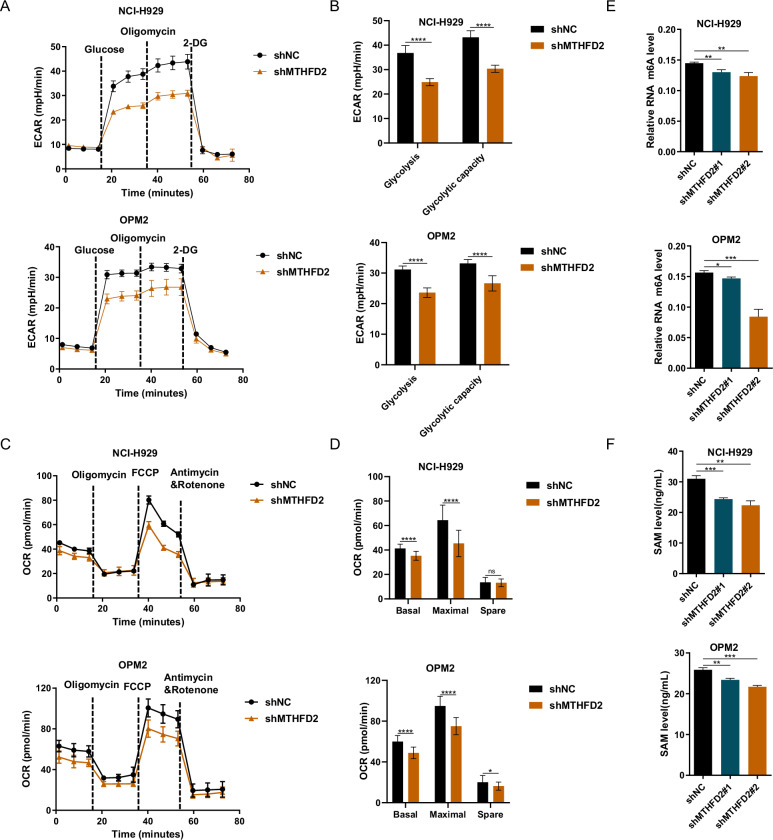
MTHFD2 knockdown leads to metabolic dysfunction in MM cells. **A** NCI-H929 and OPM2 cells stably knockdown of MTHFD2 by shRNA were used to examine ECAR via Seahorse XF Glycolysis stress test kit, performed at indicated time points by automated addition of glucose, oligomycin, 2-DG, and recorded in real time. **B** Summarized results showing glycolysis, glycolytic capacity in shNC and shMTHFD2 groups of NCI-H929 and OPM2 cells. **C** After stable MTHFD2 and NC knockdown of NCI-H929 and OPM2 cells, these cells were used to examine OCR via Seahorse XF Cell Mito Stress Test kit, performed at indicated time points by automated addition of oligomycin, FCCP, Antimycin & Rotenone and recorded in real time. **D** Summarized results were shown for basal OCR, maximal OCR and spare OCR in shNC and shMTHFD2 groups of NCI-H929 and OPM2 cells. Data represent mean ± SD from triplicate independent experiments. Student’s *t* test was used to compare two groups. **E** The total RNA m6A level of NC or MTHFD2 knockdown NCI-H929 and OPM2 cells was preformed using m6A RNA Methylation Assay kit. Data represent mean ± SD from triplicate independent experiments. **F** NCI-H929 and OPM2 cells stably knockdown of MTHFD2 by shRNA were used to examine SAM levels. The data are presented as mean ± SD. Student’s *t* test was used to compare the differences between two groups. (*P < 0.05, **P <0.01, ***P <0.001, **** *P* < 0.0001, ns not significant).

MTHFD2 has previously been reported to play an important role in the metabolism of a variety of tumors as a metabolic enzyme contributing to amino acid metabolism, nucleotide metabolism, S adenosylmethionine (SAM) cycle [37]. Glycolysis as well as oxidative phosphorylation has been reported as major modes of cellular energy supply [38]. To further elucidate the mechanism of MTHFD2 in MM, we first investigated the effect of MTHFD2 on energy expenditure patterns in MM. We used Seahorse XF analyzer to conduct experiments to detect oxygen consumption rate (OCR) and extracellular acidification rate (ECAR) of MTHFD2-knockdown NCI-H929 and OPM2 cells. We found that MTHFD2 knockdown resulted in inhibitory effects on basal glycolysis and glycolytic capacity (Fig. 6A, B). Alternatively, MTHFD2-knockdown NCI-H929 and OPM2 cells had lower basal and maximal oxidative respiration capacity compared with shNC (Fig. 6C, D). In summary, MTHFD2 knockdown significantly reduced glycolysis and mitochondrial respiratory capacity in MM cells, thereby inhibiting MM. Previous reports have shown that MTHFD2 provides important metabolic intermediates for the SAM cycle, and SAM act as a major methyl donor for RNA m6A modification, which is critical for RNA metabolism [39]. Then, we found that both the SAM level and RNA m6A level were lower in MTHFD2-knockdown NCI-H929 and OPM2 cells compared with shNC (Fig. 6E, F). These results suggest that MTHFD2 knockdown leads to metabolic dysfunction in MM cells, reflected by a decrease in glycolytic capacity, mitochondrial respiratory capacity, and SAM levels.

### Targeting MTHFD2 decreases XBP1s expression via non-metabolic functions

**Fig. 7:**
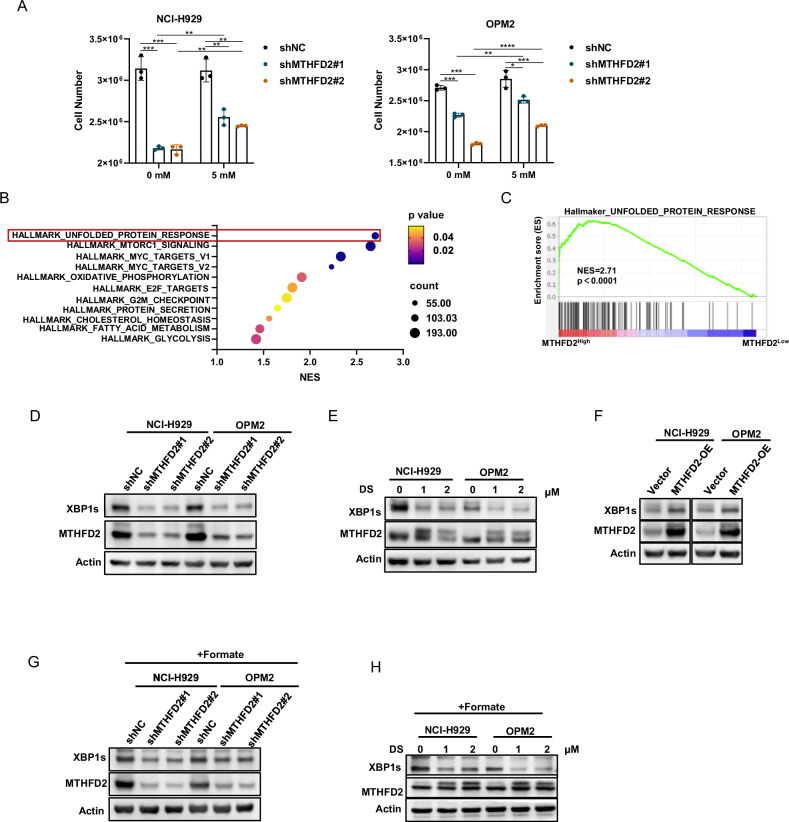
Targeting MTHFD2 decreases XBP1s expression via non-metabolic functions. **A** MTHFD2 knockdown NCI-H929 and OPM2 cells treated with 0 mM or 5 mM formate for 4 days were counted. Data represent mean ± SD from triplicate independent experiments. **B** Gene expression data grouped according to MM patients with high and low MTHFD2 expression in GSE24080 dataset showed GSEA using MSigDB hallmaker database. According to normalized enrichment score (NES) ranking, the top 11 enriched pathways were shown as bubble plots (NES > 1, *P* < 0.05). **C** The GSEA in MTHFD2 high and MTHFD2 low group of MM patients from GSE24080 dataset showing “HALLMARK_UNFOLDED_PROTEIN_RESPONSE’’, NES = 2.71,P <0.0001. **D** Western blot assay was used to detect the protein expression level of XBP1s, MTHFD2 and Actin from MTHFD2 knockdown NCI-H929 and OPM2 cells. **E** The NCI-H929 and OPM2 cells with treatment of DS in 0 μM,1 μM or 2 μM for 48 h were subjected to western blot assay to detect the protein expression level of XBP1s, MTHFD2 and Actin. **F** Western blot assay was used to detect the protein expression level of XBP1s, MTHFD2 and Actin from MTHFD2 overexpression NCI-H929 and OPM2 cells. **G** Western blot assay was performed to detect protein expression levels of XBP1s, MTHFD2 and Actin in formate 5 mM treated MTHFD2 knockdown NCI-H929 and OPM2 cells. **H** NCI-H929 and OPM2 cells were treated with 0 μM, 1 μM, 2 μM DS and formate 5 mM for 48 h. Then western blot assay was performed to detect protein expression levels of XBP1s, MTHFD2 and actin. The data are presented as mean ± SD. Student’s *t* test was used to compare the differences between two groups. (*P < 0.05, ** *P* < 0.01, *** *P* < 0.001, **** *P* < 0.0001).

Formate, which acts as an end-product of mitochondrial 1C metabolism [16, 40], supplementation of this product may have the potential to reverse MM inhibition caused by MTHFD2 knockdown. However, we found that formate supplementation did not completely restore MM cell proliferation inhibition caused by knockdown of MTHFD2 (Fig. 7A). These data illustrated that the role of MTHFD2 in MM was not limited to its enzymatic activity as a metabolic enzyme. To further explore the function of MTHFD2 in MM, we analyzed data from the GSE24080 dataset based on the expression of MTHFD2. We performed gene set enrichment analysis (GSEA) using the hallmaker gene sets from Molecular Characterization Database (MSigDB), which showed the top 11 gene sets by normalized enrichment score (NES), and the unfolded protein response (UPR) pathway ranked first (Fig. 7B, C). The UPR consists of three branches, IRE1α/XBP1s, PERK/ATF4, and ATF6, which regulates endoplasmic reticulum (ER) stress resulting from high protein synthesis in MM [41]. We then examined the major proteins of the UPR and found that protein levels of XBP1s were significantly reduced upon MTHFD2 knockdown and DS treatment in MM cells (Fig. 7D, E). However, no significant changes were observed in major proteins of other UPR branches (Fig. S3A, B). Otherwise, we found that the protein level of XBP1s was upregulated with overexpression of MTHFD2 in MM cells (Fig. 7F). Interestingly, MTHFD2 knockdown by shRNA and DS treatment of MM cells both resulted in downregulation of XBP1s expression, which was not restored by formate. These findings suggest that XBP1s protein alterations resulting from targeting MTHFD2 are not through its metabolic function.

### Targeting MTHFD2 synergizes with bortezomib against MM in vitro and in vivo

**Fig. 8:**
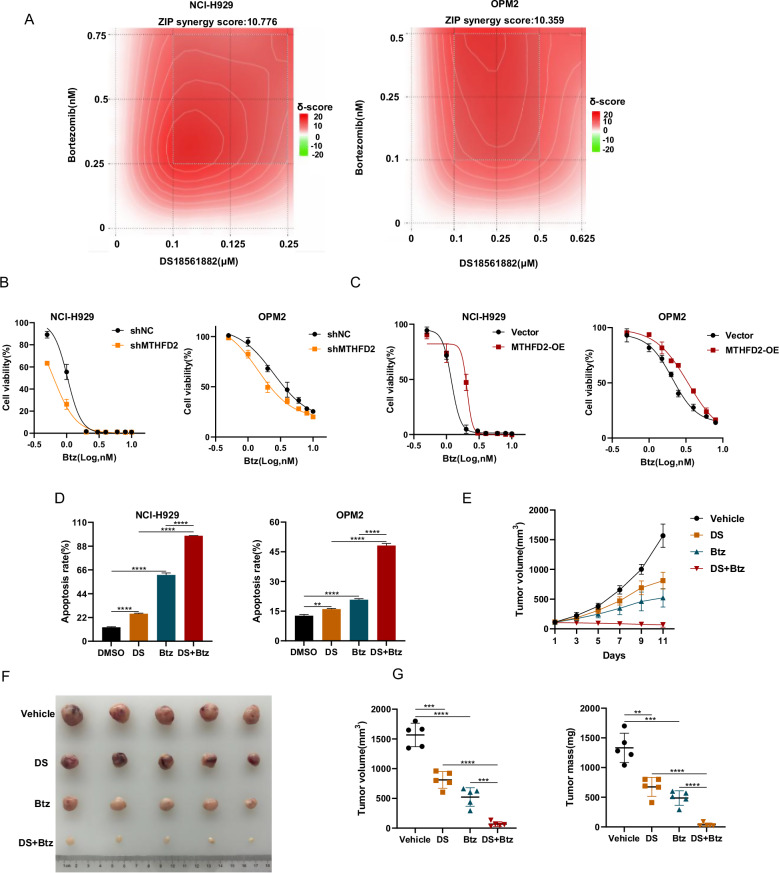
Targeting MTHFD2 synergizes with bortezomib against MM in vitro and in vivo. **A** CCK-8 assay was used to detect cell viability in NCI-H929 and OPM2 cells treated with bortezomib and DS at the indicated concentrations for 48 h, and then handled with “SynergyFinder” to calculate the ZIP synergy score representing the combined effect of the two drugs. **B**, **C** The cell viability of MTHFD2 knockdown or overexpression NCI-H929 and OPM2 cells treated with bortezomib at the indicated concentrations for 48 h was examined by CCK-8 assay. Data represent mean ± SD from triplicate independent experiments. **D** The apoptosis rate of NCI-H929 treated with DMSO, DS 1.5 μM, Btz 2 nM or DS 1.5 μM +Btz 2 nM for 48 h and OPM2 cells treated with DMSO, DS 0.5 μM, Btz 2 nM or DS 0.5 μM +Btz 2 nM for 48 h was examined by flow cytometry, then results as bar graph were calculated percentages of Annexin-V positive cell populations from three independent experiments. **E** NCI-H929 were subcutaneously injected into NOG mice to establish MM xenograft mouse models. Then mice were randomly divided into four groups (vehicle, DS group, Btz group and DS+Btz group) respectively (*n* = 5 per group). Tumor volumes represented by line graphs were recorded every other day after treatment. **F** Tumors from these mice were dissected and photographed 11 days after administration of vehicle, DS (100 mg/kg/day, d1-d11, oral gavage), Btz (0.5 mg/kg, d1, d4, d8, d11, intravenous injections) or DS+Btz. **G** Tumor volumes and weights were then assessed for the four groups described above. These mouse experiments were repeated once. Student’s *t* test was used to compare two groups. (**P < 0.01, ***P < 0.001, ****P < 0.0001).

Bortezomib (Btz) acts primarily on the proteasome 26S subunit, leading to increased ER stress, which activates excessive UPR and ultimately MM cell death [27, 28]. Otherwise, Btz causes metabolic disorders in MM cells by decreasing mitochondrial respiratory function [29]. Our findings suggest that MTHFD2 inhibited mitochondrial respiration and impacted the UPR in MM cells, so we hypothesized that targeting MTHFD2 may be useful in combination with Btz for the treatment of MM. Then, we performed CCK-8 assays and found that DS and Btz had synergistic inhibitory effects on MM proliferation (Fig. 8A). Otherwise, as shown in Fig. 8B, C, the sensitivity of MM cells to Btz increased with knockdown of MTHFD2 and decreased with overexpression of MTHFD2. We further treated MM cells with DS, Btz, or DS + Btz for 48 h and found that the apoptotic rate in the combination group was significantly higher than that in the monotherapy groups (Figs. 8D and S4A). We then explored the combined effect of DS and Btz in terms of metabolism. We found that DS inhibited glycolysis and mitochondrial respiratory capacity, which was parallel to the phenomena caused by MTHFD2 knockdown. DS synergized with Btz to decrease the glycolysis and mitochondrial respiratory capacity(Fig. S4B–E).We then used NCI-H929 cells to construct MM mouse xenograft subcutaneous tumor model and found that mice in the combination group had significantly smaller tumors and lower proliferation rate than those in the monotherapy groups, and mice in the combination group showed no significant decrease in body weight (Figs. 8E–G and S4G). Consistently, immunohistochemistry of tumors in the DS+Btz group showed lower Ki67 and significantly increased cleaved-caspase3 (Fig. S4). In conclusions, DS cooperates with bortezomib against MM in vitro and in vivo.

## Discussion

In the present study, we found that MTHFD2 is highly expressed in MM and is associated with poor prognosis. Targeting MTHFD2 significantly inhibits MM proliferation, promotes apoptosis and causes G0/G1 phase cycle arrest in vitro. And targeting MTHFD2 also has anti-MM effects in vivo. As an important enzyme in 1C metabolism, knockdown of MTHFD2 could significantly cause metabolic changes in MM, mainly including inhibition of MM glycolysis, depressed oxidative phosphorylation ability, and decreased level of SAM. SAM, as a methyl donor modified by m6A, plays an important role in the metabolism, localization, and degradation of RNA [39, 42]. MTHFD2 therefore plays an important role in MM malignancy.

In addition, it has been reported that MTHFD2 is present in the nucleus and interacts with cyclin-dependent kinase 2 (CDK2), and plays an important role in bladder cancer through non-metabolic functions [24]. Another study showed that MTHFD2 binds to DNA replication sites in the nucleus and has crucial effect in the proliferation of tumors [43]. Thus, inhibition of cell proliferation caused by MTHFD2 knockdown in MM cells could not be totally reversed by formate, a metabolic end-product of 1 C metabolism in mitochondria, suggesting that MTHFD2 also acts as a non-metabolizing enzyme. Further bioinformatics analysis showed that MTHFD2 mainly affected the UPR of MM cells, and MM cells required UPR to regulate ER stress due to their continuous ER stress status, so as to achieve malignant proliferation. We then demonstrated the nonmetabolic functions of MTHFD2 by the downregulation of XBP1s, an important part of UPR, via targeting MTHFD2. XBP1s was particularly important for MM as well as plasma cell differentiation, and it has been reported that IREα/XBP1 can be used as an important therapeutic target for MM [35, 44]. PIs have been shown to elicit terminal UPR and caused metabolic dysfunction by suppressing mitochondrial respiratory capacity [28, 29]. Therefore, we supposed that MTHFD2 was associated with the drug sensitivity of Btz. It was found that the sensitivity to Btz increased and decreased after knockdown and overexpression of MTHFD2. DS synergized with Btz to decrease the glycolysis and mitochondrial respiratory capacity. Moreover, we treated MM cells and MM mice with DS, a MTHFD2 inhibitor, in combination with Btz, and found the inhibitor had significant synergistic anti-MM effects in vivo and in vitro. Although our above findings demonstrate the synergistic anti-MM effects of DS and Btz in cell metabolism, cell proliferation and apoptosis. But the limitation of our study is that no further experiments using Btz resistant MM cell lines were performed to demonstrate the synergistic effect of the two drugs.

MTHFD2 has been reported to affect the protein modulation of HIF1α by regulating m6A modification in renal cell carcinoma, which leads to decreased glycolytic capacity of renal tumor cells [22]. When targeting MTHFD2 in MM, more researches are needed to investigate the particular genes affected by m6A modification, and the following changes caused by m6A modification level changes. In addition to targeting the intracellular localization of MTHFD2, further exploration is needed, and in our study, we found that the knockdown and application of DS can lead to G0/G1 arrest in MM cells. We also need to further explore whether MTHFD2 has some correlation with cell cycle-related proteins. Otherwise, we found that the MTHFD2 inhibitor DS killed CD138+ MM cells relatively specifically, and had low cytotoxicity to normal human PBMCs as well as CD138-MM cells. Therefore, it provides certain value for the clinical use of DS in the later stage.

It has already been reported that MTHFD2 primarily affects the glycosylation modification of c-Myc, resulting in changes of the half-life of c-Myc protein, which ultimately causes immune escape in pancreatic cancer by up-regulating PD-L1 [45]. Previous studies have shown that c-Myc is a transcription factor for MTHFD2 in AML and glioblastoma [23, 46]. C-Myc plays an important role in the pathogenesis of MM [47]. We next need to investigate whether the high expression level of MTHFD2 in MM is associated with c-Myc, and the relationship between c-Myc and MTHFD2 in MM. In addition, MTHFD2 has been shown to be associated with autoimmune diseases by regulating the fate and function of T cells [48], providing a new theoretical basis for the research about the subsequent clinical treatment of MM and immunotherapy resistance.

## Conclusions

In conclusion, targeting MTHFD2 alters metabolic homeostasis and influences UPR through nonmetabolic functions in MM cells. Otherwise, targeting MTHFD2 and co-administration with bortezomib have significant anti-MM effects in vitro and in vivo. The above findings suggest that MTHFD2 is a valuable target for MM therapy.

## Materials and methods

### Patient samples

Bone marrow mononuclear cells (BMMCs) from MM patients and peripheral blood mononuclear cells (PBMCs) from healthy donors were isolated by Ficoll-Paque Plus (Sigma-Aldrich, St. Louis, MO) using density gradient centrifugation. CD138-positive plasma cells from BMMCs were then isolated by anti-CD138 beads (Miltenyi Biotec, Bergisch Gladbach, Germany) according to the manufacturer ‘s instructions.

### Plasmid construction and lentiviral packaging

The Sequences encoding shRNAs against MTHFD2 were cloned into lentiviral pLKO.1-puro vector. MTHFD2 overexpression plasmids were constructed by inserting MTHFD2 cDNA fragment obtained by PCR into the pLVX-puro vector using ClonExpress II One Step Cloning Kit (Vazyme, Nanjing, China). Lentiviral vectors were co-transfected into HEK293T cells with packaging plasmids psPAX2 and pMD2G. Infectious lentiviruses were harvested and filtered through 0.45 μm PVDF filters. The shRNA sequences of MTHFD2 were list as following: shMTHFD2#1: 5′-CGAGAAGTGCTGAAGTCTAAA-3′ and shMTHFD2#2: 5′-GCAGTTGAAGAAACATACAAT-3′.

### Animal experiments

All animal experiments were conducted in according to the ARRIVE guidelines and approved by the Animal Care and Use Committee of Shanghai Jiao Tong University School of Medicine. The 4-week NOG mice were purchased from Vital River Laboratory Animal Technology Co., Ltd. (Beijing, China). MM cells were infected with lentivirus of shNC and shMTHFD2 to construct stable knockdown of MTHFD2 or negative control cells, then 3 × 10^6^ cells were resuspended in PBS and Corning Matrigel and subcutaneously injected into the right flank of NOG mice. Three weeks later, mice were euthanized under carbon dioxide anesthesia and tumor tissues were weighed and then fixed with 4% formalin solution or stored at −80 °C for the following experiments.

3 × 10^6^ NCI-H929 cells or OPM2 cells were mixed with Matrigel and subcutaneously injected into the right flank of the mice. When tumors were measurable (day 1), mice were randomized into vehicle group and DS18561882 treatment group (5 mice per group). In the treatment groups, mice received DS18561882 (oral gavage) at 150 mg/kg once a day for 11 days. For the two-drug combination experiment, 3 × 10^6^ NCI-H929 cells were mixed with Matrigel and subcutaneously injected into the right flank of the mice. When tumors were measurable (Day 1), mice were randomized into vehicle group, DS18561882 treatment group, bortezomib treatment group and combination group. In the treatment groups, mice received DS18561882 (oral gavage) at 100 mg/kg once a day for 11 days, or/and bortezomib at 0.5 mg/kg intravenously (Day 1, 4, 8 and 11). For the above drug experiments, tumor size and mice weights were measured every 2 days by a caliper. The tumor volume (TV) was calculated using the formula TV = Length × Width^2^/2. At the end of the experiment, mice were sacrificed by carbon dioxide asphyxiation and tumor tissues were weighed. and then fixed with 4% formalin solution or stored at −80 °C for the following experiments.

### Statistical analysis

Data are shown as the mean ± standard deviation (SD) and two-tailed unpaired or paired Student’s *t* tests were applied to compare differences between two groups. Two-way analysis of variance (ANOVA) was used for multiple groups analysis. Overall survival of MM patients was evaluated using the Kaplan–Meier method and curves were compared using the log-rank (Man-tel-Cox) test. The IC50 value was calculated based on the dose–response curve using GraphPad Prism 9.0 software. Statistical differences were indicated by *P < 0.05, **P < 0.01, and ***P < 0.001, ****P < 0.0001.

## Supplementary information

supplementary materials and methods and supplementary figure legends

supplementary figures

original western blots

## Data Availability

The data and materials are available from the corresponding author on reasonable request.

## References

[1] Siegel RL, Miller KD, Wagle NS, Jemal A. Cancer statistics, 2023. CA Cancer J Clin. 2023;73:17–48. 36633525 10.3322/caac.21763

[2] Malard F, Neri P, Bahlis NJ, Terpos E, Moukalled N, Hungria VTM, et al. Multiple myeloma. Nat Rev Dis Prim. 2024;10:45. 38937492 10.1038/s41572-024-00529-7

[3] Xiong S, Chng WJ, Zhou J. Crosstalk between endoplasmic reticulum stress and oxidative stress: a dynamic duo in multiple myeloma. Cell Mol Life Sci. 2021;78:3883–906. 33599798 10.1007/s00018-021-03756-3 PMC8106603

[4] Panaroni C, Fulzele K, Mori T, Siu KT, Onyewadume C, Maebius A, et al. Multiple myeloma cells induce lipolysis in adipocytes and uptake fatty acids through fatty acid transporter proteins. Blood. 2022;139:876–88. 34662370 10.1182/blood.2021013832 PMC8832479

[5] Gu Z, Xia J, Xu H, Frech I, Tricot G, Zhan F. NEK2 promotes aerobic glycolysis in multiple myeloma through regulating splicing of pyruvate kinase. J Hematol Oncol. 2017;10:17. 28086949 10.1186/s13045-017-0392-4 PMC5237262

[6] Zhu Y, Jian X, Chen S, An G, Jiang D, Yang Q, et al. Targeting gut microbial nitrogen recycling and cellular uptake of ammonium to improve bortezomib resistance in multiple myeloma. Cell Metab. 2024;36:159–75.e158. 38113887 10.1016/j.cmet.2023.11.019

[7] Wu Y, Luo Y, Yao X, Shi X, Xu Z, Re J, et al. KIAA1429 increases FOXM1 expression through YTHDF1-mediated m6A modification to promote aerobic glycolysis and tumorigenesis in multiple myeloma. Cell Biol Toxicol. 2024;40:58. 39060874 10.1007/s10565-024-09904-2 PMC11282141

[8] Huang H, Chen Y, Li Y, Zheng X, Shu L, Tian L, et al. Cytidine triphosphate synthase 1-mediated metabolic reprogramming promotes proliferation and drug resistance in multiple myeloma. Heliyon. 2024;10:e33001. 39050461 10.1016/j.heliyon.2024.e33001 PMC11268195

[9] Voorhees PM, Kaufman JL, Laubach J, Sborov DW, Reeves B, Rodriguez C, et al. Daratumumab, lenalidomide, bortezomib, and dexamethasone for transplant-eligible newly diagnosed multiple myeloma: the GRIFFIN trial. Blood. 2020;136:936–45. 32325490 10.1182/blood.2020005288 PMC7441167

[10] Attal M, Lauwers-Cances V, Hulin C, Leleu X, Caillot D, Escoffre M, et al. Lenalidomide, bortezomib, and dexamethasone with transplantation for myeloma. N Engl J Med. 2017;376:1311–20. 28379796 10.1056/NEJMoa1611750 PMC6201242

[11] Dimopoulos MA, Terpos E, Boccadoro M, Delimpasi S, Beksac M, Katodritou E, et al. Daratumumab plus pomalidomide and dexamethasone versus pomalidomide and dexamethasone alone in previously treated multiple myeloma (APOLLO): an open-label, randomised, phase 3 trial. Lancet Oncol. 2021;22:801–12. 34087126 10.1016/S1470-2045(21)00128-5

[12] Cortes-Selva D, Perova T, Skerget S, Vishwamitra D, Stein S, Boominathan R, et al. Correlation of immune fitness with response to teclistamab in relapsed/refractory multiple myeloma in the MajesTEC-1 study. Blood. 2024;144:615–28. 38657201 10.1182/blood.2023022823 PMC11347796

[13] Munshi NC, Anderson LD Jr, Shah N, Madduri D, Berdeja J, Lonial S, et al. Idecabtagene vicleucel in relapsed and refractory multiple myeloma. N Engl J Med. 2021;384:705–16. 33626253 10.1056/NEJMoa2024850

[14] Mailankody S, Devlin SM, Landa J, Nath K, Diamonte C, Carstens EJ, et al. GPRC5D-targeted CAR T cells for myeloma. N Engl J Med. 2022;387:1196–206. 36170501 10.1056/NEJMoa2209900 PMC10309537

[15] Ducker GS, Rabinowitz JD. One-carbon metabolism in health and disease. Cell Metab. 2017;25:27–42. 27641100 10.1016/j.cmet.2016.08.009 PMC5353360

[16] Locasale JW. Serine, glycine and one-carbon units: cancer metabolism in full circle. Nat Rev Cancer. 2013;13:572–83. 23822983 10.1038/nrc3557 PMC3806315

[17] Nilsson R, Jain M, Madhusudhan N, Sheppard NG, Strittmatter L, Kampf C, et al. Metabolic enzyme expression highlights a key role for MTHFD2 and the mitochondrial folate pathway in cancer. Nat Commun. 2014;5:3128. 24451681 10.1038/ncomms4128 PMC4106362

[18] Di Pietro E, Sirois J, Tremblay ML, MacKenzie RE. Mitochondrial NAD-dependent methylenetetrahydrofolate dehydrogenase-methenyltetrahydrofolate cyclohydrolase is essential for embryonic development. Mol Cell Biol. 2002;22:4158–66. 12024029 10.1128/MCB.22.12.4158-4166.2002 PMC133862

[19] Jain M, Nilsson R, Sharma S, Madhusudhan N, Kitami T, Souza AL, et al. Metabolite profiling identifies a key role for glycine in rapid cancer cell proliferation. Science. 2012;336:1040–4. 22628656 10.1126/science.1218595 PMC3526189

[20] Mo H-Y, Wang R-B, Ma M-Y, Zhang Y, Li X-Y, Wen W-R, et al. MTHFD2-mediated redox homeostasis promotes gastric cancer progression under hypoxic conditions. Redox Rep. 2024;29:2345455. 38723197 10.1080/13510002.2024.2345455 PMC11086033

[21] Ju HQ, Lu YX, Chen DL, Zuo ZX, Liu ZX, Wu QN, et al. Modulation of redox homeostasis by inhibition of MTHFD2 in colorectal cancer: mechanisms and therapeutic implications. J Natl Cancer Inst. 2019;111:584–96. 30534944 10.1093/jnci/djy160 PMC6579745

[22] Green NH, Galvan DL, Badal SS, Chang BH, LeBleu VS, Long J, et al. MTHFD2 links RNA methylation to metabolic reprogramming in renal cell carcinoma. Oncogene. 2019;38:6211–25. 31289360 10.1038/s41388-019-0869-4 PMC8040069

[23] Pikman Y, Puissant A, Alexe G, Furman A, Chen LM, Frumm SM, et al. Targeting MTHFD2 in acute myeloid leukemia. J Exp Med. 2016;213:1285–306. 27325891 10.1084/jem.20151574 PMC4925018

[24] Liu X, Liu S, Piao C, Zhang Z, Zhang X, Jiang Y, et al. Non-metabolic function of MTHFD2 activates CDK2 in bladder cancer. Cancer Sci. 2021;112:4909–19. 34632667 10.1111/cas.15159 PMC8645701

[25] Nishimura T, Nakata A, Chen X, Nishi K, Meguro-Horike M, Sasaki S, et al. Cancer stem-like properties and gefitinib resistance are dependent on purine synthetic metabolism mediated by the mitochondrial enzyme MTHFD2. Oncogene. 2019;38:2464–81. 30532069 10.1038/s41388-018-0589-1 PMC6484769

[26] Zhao R, Feng T, Gao L, Sun F, Zhou Q, Wang X, et al. PPFIA4 promotes castration-resistant prostate cancer by enhancing mitochondrial metabolism through MTHFD2. J Exp Clin Cancer Res. 2022;41:125. 35382861 10.1186/s13046-022-02331-3 PMC8985307

[27] Lee AH, Iwakoshi NN, Anderson KC, Glimcher LH. Proteasome inhibitors disrupt the unfolded protein response in myeloma cells. Proc Natl Acad Sci USA. 2003;100:9946–51. 12902539 10.1073/pnas.1334037100 PMC187896

[28] Obeng EA, Carlson LM, Gutman DM, Harrington WJ Jr, Lee KP, Boise LH. Proteasome inhibitors induce a terminal unfolded protein response in multiple myeloma cells. Blood. 2006;107:4907–16. 16507771 10.1182/blood-2005-08-3531 PMC1895817

[29] Wang Q, Zhao D, Xian M, Wang Z, Bi E, Su P, et al. MIF as a biomarker and therapeutic target for overcoming resistance to proteasome inhibitors in human myeloma. Blood. 2020;136:2557–73. 32582913 10.1182/blood.2020005795 PMC7714094

[30] Kubicki T, Bednarek K, Kostrzewska-Poczekaj M, Luczak M, Lewandowski K, Gil L, et al. Bortezomib- and carfilzomib-resistant myeloma cells show increased activity of all three arms of the unfolded protein response. Am J Cancer Res. 2022;12:3280–93. 35968359 PMC9360248

[31] Todd DJ, Lee AH, Glimcher LH. The endoplasmic reticulum stress response in immunity and autoimmunity. Nat Rev Immunol. 2008;8:663–74. 18670423 10.1038/nri2359

[32] Yoshida H, Matsui T, Yamamoto A, Okada T, Mori K. XBP1 mRNA is induced by ATF6 and spliced by IRE1 in response to ER stress to produce a highly active transcription factor. Cell. 2001;107:881–91. 11779464 10.1016/s0092-8674(01)00611-0

[33] Lee AH, Iwakoshi NN, Glimcher LH. XBP-1 regulates a subset of endoplasmic reticulum resident chaperone genes in the unfolded protein response. Mol Cell Biol. 2003;23:7448–59. 14559994 10.1128/MCB.23.21.7448-7459.2003 PMC207643

[34] Nikesitch N, Tao C, Lai K, Killingsworth M, Bae S, Wang M, et al. Predicting the response of multiple myeloma to the proteasome inhibitor Bortezomib by evaluation of the unfolded protein response. Blood Cancer J. 2016;6:e432. 27284736 10.1038/bcj.2016.40 PMC5141355

[35] Carrasco DR, Sukhdeo K, Protopopova M, Sinha R, Enos M, Carrasco Daniel E, et al. The differentiation and stress response factor XBP-1 drives multiple myeloma pathogenesis. Cancer Cell. 2007;11:349–60. 17418411 10.1016/j.ccr.2007.02.015 PMC1885943

[36] Kawai J, Toki T, Ota M, Inoue H, Takata Y, Asahi T, et al. Discovery of a potent, selective, and orally available MTHFD2 inhibitor (DS18561882) with in vivo antitumor activity. J Med Chem. 2019;62:10204–20. 31638799 10.1021/acs.jmedchem.9b01113

[37] Ramos L, Henriksson M, Helleday T, Green AC. Targeting MTHFD2 to exploit cancer-specific metabolism and the DNA damage response. Cancer Res. 2024;84:9–16. 37922465 10.1158/0008-5472.CAN-23-1290

[38] Vander Heiden MG, Cantley LC, Thompson CB. Understanding the Warburg effect: the metabolic requirements of cell proliferation. Science. 2009;324:1029–33. 19460998 10.1126/science.1160809 PMC2849637

[39] Chen X-Y, Zhang J, Zhu J-S. The role of m6A RNA methylation in human cancer. Mol Cancer. 2019;18:103. 31142332 10.1186/s12943-019-1033-z PMC6540575

[40] Christensen KE, Mackenzie RE. Mitochondrial methylenetetrahydrofolate dehydrogenase, methenyltetrahydrofolate cyclohydrolase, and formyltetrahydrofolate synthetases. Vitam Horm. 2008;79:393–410. 18804703 10.1016/S0083-6729(08)00414-7

[41] Chen X, Cubillos-Ruiz JR. Endoplasmic reticulum stress signals in the tumour and its microenvironment. Nat Rev Cancer. 2020;21:71–88. 33214692 10.1038/s41568-020-00312-2 PMC7927882

[42] Mahmoud AM, Ali MM. Methyl donor micronutrients that modify DNA methylation and cancer outcome. Nutrients. 2019;11:608. 30871166 10.3390/nu11030608 PMC6471069

[43] Gustafsson Sheppard N, Jarl L, Mahadessian D, Strittmatter L, Schmidt A, Madhusudan N, et al. The folate-coupled enzyme MTHFD2 is a nuclear protein and promotes cell proliferation. Sci Rep. 2015;5:15029. 26461067 10.1038/srep15029 PMC4602236

[44] Mimura N, Fulciniti M, Gorgun G, Tai YT, Cirstea D, Santo L, et al. Blockade of XBP1 splicing by inhibition of IRE1α is a promising therapeutic option in multiple myeloma. Blood. 2012;119:5772–81. 22538852 10.1182/blood-2011-07-366633 PMC3382937

[45] Shang M, Yang H, Yang R, Chen T, Fu Y, Li Y, et al. The folate cycle enzyme MTHFD2 induces cancer immune evasion through PD-L1 up-regulation. Nat Commun. 2021;12:1940. 33782411 10.1038/s41467-021-22173-5 PMC8007798

[46] Zhu Z, Kiang KM, Li N, Liu J, Zhang P, Jin L, et al. Folate enzyme MTHFD2 links one-carbon metabolism to unfolded protein response in glioblastoma. Cancer Lett. 2022;549:215903. 36089117 10.1016/j.canlet.2022.215903

[47] van de Donk N, Pawlyn C, Yong KL. Multiple myeloma. Lancet. 2021;397:410–27. 33516340 10.1016/S0140-6736(21)00135-5

[48] Sugiura A, Andrejeva G, Voss K, Heintzman DR, Xu X, Madden MZ, et al. MTHFD2 is a metabolic checkpoint controlling effector and regulatory T cell fate and function. Immunity. 2022;55:65–81.e69. 34767747 10.1016/j.immuni.2021.10.011 PMC8755618

