# Supplementary material for: Targeting MTHFD2 alters metabolic homeostasis and synergizes with bortezomib to inhibit multiple myeloma
Source: Cell Death Discov. 2025 Apr 25;11:201. doi: 10.1038/s41420-025-02498-6 (PMC12032361; doi:10.1038/s41420-025-02498-6)
Supplement: Supplementary file 1 — supplementary materials and methods and supplementary figure legends [file 41420_2025_2498_MOESM1_ESM.docx]

**Supplementary information**

**Title:**

**Targeting MTHFD2 alters metabolic homeostasis and synergizes with bortezomib to inhibit multiple myeloma**

**Supplementary information includes supplementary materials and methods and supplementary figure legends**

**Supplementary materials and methods**

**Cell culture and reagents**

The human MM cell line NCI-H929, OPM2, MM.1S, U266, LP-1, RPMI 8266 and HEK293T were purchased from American Type Culture Collection (Manassas, VA, USA). All cells were cultured in a humidified 5% C0_2_ incubator at 37℃. Cell lines were routinely tested negative for mycoplasma contamination.MM cells were cultured in RPMI 1640 medium. HEK293T cells were cultured in DMEM medium. All cells were supplemented with 10% fetal bovine serum (Sigma Aldrich, St.Louis, MO) and 100 IU/mL penicillin (Invitrogen) and 100 μg/mL streptomycin (YEASEN, China) in the above medium.

DS18561882, bortezomib was purchased from MCE (MedChemExpress, USA) and dissolved in dimethyl sulfoxide (Sigma, St. Louis, MO) and stored at − 20° C in the dark until use.

**Bioinformatics**

The survival data and gene expression of Multiple Myeloma Research Foundation (MMRF) CoMMpass study were downloaded from the University of California Santa Cruz Xena database (https://xena.ucsc.edu). The gene expression data of GSE6477, GSE46816 were obtained from Gene Expression Omnibus (GEO, http://www.ncbi.nlm.nih.gov/geo/). Enrichment analysis was performed using the GSEA website MSIGDB database (http://software.broadin stitute.org/gsea/msigdb). FDR q-value <0.25, nominal p-value<0.05 and |NES|>1 was identified as significantly enriched.

**Quantitative reverse transcription real-time PCR (qRT-PCR)**

Total cellular RNA extraction was performed using TRIzol reagent (Invitrogen, Carlsbad, CA) and then converted to cDNA using Evo M-MLV RT Kit with gDNA Clean for qPCR kit (Accurate Biology, Changsha, China). The cDNA was used as a template for qRT-PCR using SYBR Green Premix Pro Taq HS qPCR Kit (Accurate Biology, Changsha, China). The expression levels of all gene were normalized to β-actin and calculated using the 2^−ΔΔCt^ formula. The primers for qRT-PCR were listed as following: MTHFD2-F: TGGCTGCGACTTCTCTAATG, MTHFD2-R: CCTTCCAGAAATGACAACAGC; β-actin-F: CATGTACGTTGCTATCCAGGC, β-actin-R: CTCCTTAATGTCACGCACGAT.

**Cell proliferation assay**

Cell proliferation assays were performed using the Cell Counting Kit-8 (CCK-8) kit (New Cell and Molecular Biotech, Suzhou, China). MM cells were seeded in triplicate in 96-well plates. CCK-8 reagent was added to the corresponding drug treatment for 48 hours or at the corresponding testing time point, and absorbance at 450 nm was measured using a microplate reader (BioTek Instruments, Winooski, VT). The combined effect of the two drugs using Synergyfinder ( https://synergyfinder.fimm.fi/)[35],ZIP synergy score > 10, between -10 and 10, and < 10, indicating synergism, additive effect, and antagonism, respectively.

**Apoptosis assay**

After MM cells collection and washing with phosphate buffer saline (PBS), apoptosis assay was measured by flow cytometry using Annexin V and PI detection kits (BD Biosciences, San Diego, CA, USA). Apoptotic cells included early (Annexin V positive and PI negative) and late (Annexin V positive and PI positive) apoptosis cells. Results were analyzed by FlowJo10.0 software.

**Detection of cell cycle**

MM cells were collected, washed with ice-cold PBS, then fixed in 70% ethanol at -20 ° C for not less than 24 hours. Then the cells incubated in the dark with PI/RNase staining buffer (BD Biosciences). Fluorescence of stained cells was measured using a CytoFLEX cytometer (Beckman, Brea, CA). Cell cycle was determined by PI staining assay. The distribution of each cell cycle was analyzed by ModFit software.

**Western blot**

Cells are collected, washed with ice-cold PBS, and lysed in in 1× SDS. Equal amounts of protein extract are electrophoresed on sodium dodecyl sulfate polyacrylamide gels and transferred to ni-trocellulose membranes. After blocking with 5% skimmed milk, the membrane was incubated with the primary antibody overnight at 4°C and then with horseradish peroxidase (HRP)-conjugated secondary antibody. The signals were detected using a chemiluminescent phototope -HRP kit (Cell Signaling Technology, Danvers, MA) according to the manufacturer 's instructions.

Antibodies against MTHFD2 (catalog, 12270-1-AP), XBP1s (catalog, 24868-1-AP), GRP78 (catalog, 11587-1-AP), ATF6 (catalog, 24169-1-AP), CHOP (catalog, 15204-1-AP), CDK4 (catalog, 66950-1-Ig), PARP1 (catalog, 13371-1-AP), Caspase3 (catalog, 19677-1-AP), cyclinD1 (catalog, 60186-1-Ig), β-actin (catalog, 66009-1-Ig).

**Human SAM ELISA assay**

The detection of SAM levels of MM cells was performed using Human S-adenosylmethionine (SAM) ELISA assay kit (Yanqi biotechnology, Shanghai, China). Briefly, after capture antibody solution of SAM was coated on the wells, then the cell culture medium supernatant of MM cells in triplicates and standard were added. According to the manufacturer's protocol of this kit, the secondary detection antibody solution was added. In the end, the SAM levels were quantified according to the absorbance at 450 nm measured using a microplate reader (BioTek Instruments, Winooski, VT), and then calculated from a standard curve.

**Seahorse assay**

The detection of oxygen consumption rate (OCR) and extracellular acidification rate (ECAR) were performed using Seahorse XF analyzer (Agilent, California, CA, USA). MM cells (5×10^5^ per well) suspended in Seahorse XF base medium (supplemented with 1 mM sodium pyruvate,10 mM glucose, 2 mM L-glutamine, pH 7.4) were in six replicates and then transferred into a pretreated Cell-Tak-coated XF 96-well. Oligomycin (1.5 µM), FCCP (1.5 µM) and rotenone/antimycin A (0.5 µM) were injected as indicated and the OCR (pmol/min) was measured in real time according to manufacturer's protocol for the XF Cell Mito Stress Test kit (Agilent, California, CA, USA). Glucose (10 mM), oligomycin (1.5 μM) and 2-deoxy-glucose (2-DG) (50 mM) were injected as indicated and the ECAR (mpH/min) was measured in real time according to manufacturer's protocol for XF Glycolysis stress test kit (Agilent, California, CA, USA).

**RNA m6A methylation assay**

The total RNA m6A methylation measurement of MM cells was performed using m6A RNA Methylation Assay kit (ab185912, Abcam, Cambridge, UK). According to the manufacturer's protocol of this kit, 200 ng of RNA from MM cells and m6A standard was coated on the test wells, and then the capture antibody solution and the secondary detection antibody solution were added. Colorimetric m6A levels were quantified according to the absorbance at 450 nm measured using a microplate reader (BioTek Instruments, Winooski, VT), and then calculated from a standard curve.

**Supplementary Figure legends**

**Supplementary Figure.1 DS induces apoptosis and causes G0/G1 arrest in MM cells**

(A) Annexin V/PI double staining flow cytometry was used to detect apoptosis in NCI-H929 and OPM2 cells treated with DS 0 μM, 1 μM, and 2 μM for 48 h. (B) After treatment with DS 0 μM, 1 μM, and 2 μM for 48 h, the cell cycles of NCI-H929 and OPM2 cells were examined. Results were presented as peak plots using Modfit.

**Supplementary Figure.2 DS exhibits anti-MM effects in vivo**

(A) Body weights of mice were recorded every other day after vehicle or DS administration until day 11. Student's t-test was used for two-group analysis on day 11(ns: no significance).

**Supplementary Figure.3 MTHFD2 knockdown do not cause significant changes in other UPR proteins.**

(A, B) Western blot assays showed protein levels in NCI-H929 and OPM2 cells following MTHFD2 knockdown, including GRP78, CHOP, ATF6, MTHFD2, Actin.

**Supplementary Figure.4 DS cooperates with bortezomib against MM cells**

(A) NCI-H929 cells were exposed to either 0 μM or 1. 5 μM of DS in the presence or absence of Btz 2 nM for 48 h. OPM2 cells were exposed to either 0 μM or 0.5 μM of DS in the presence or absence of Btz 2 nM for 48 h. Apoptosis was then detected by flow cytometry with Annexin V/PI double staining. (B) NCI-H929 and OPM2 cells were treated with DMSO, DS (0.5 μM), Btz (1 nM）or DS(0.5 μM)+BTZ (1 nM) for 24 h, these cells were used to examine OCR via Seahorse XF Cell Mito Stress Test kit, performed at indicated time points by automated addition of oligomycin, FCCP, Antimycin & Rotenone and recorded in real time. (C) Summarized results were shown for basal OCR, maximal OCR and spare OCR in the above cells. (D) NCI-H929 and OPM2 cells were treated with DMSO, DS (0.5 μM), Btz (1 nM）or DS(0.5 μM)+BTZ (1 nM) for 24 h, these were used to examine ECAR via Seahorse XF Glycolysis stress test kit, performed at indicated time points by automated addition of glucose, oligomycin, 2-DG, and recorded in real time. (E) Summarized results showing glycolysis, glycolytic capacity in these cells. (F) Body weights of mice were recorded every other day after treatment until day 11. (G) Paraffin-embedded tumor sections of vehicle, DS group, Btz group and DS+Btz group were stained with hematoxylin and eosin (HE), Ki67 and cleaved caspase-3 antibodies (scale bar 100 um). Data are presented as mean ± SD. Two-way analysis of variance (ANOVA) was used for multiple groups analysis. (* P＜0.05，** P＜0.01， **** P＜0.0001).
