# Supplementary material for: Targeting MTHFD2 alters metabolic homeostasis and synergizes with bortezomib to inhibit multiple myeloma
Source: Cell Death Discov. 2025 Apr 25;11:201. doi: 10.1038/s41420-025-02498-6 (PMC12032361; doi:10.1038/s41420-025-02498-6)

Supplementary Figure 1

A

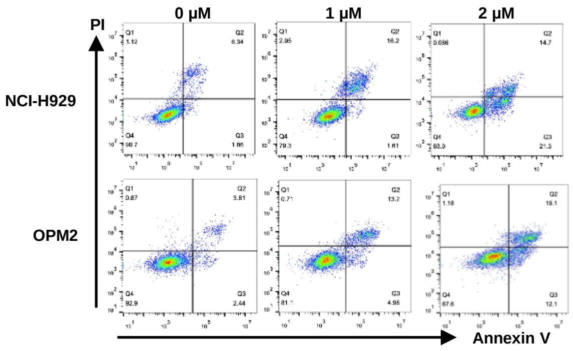

B

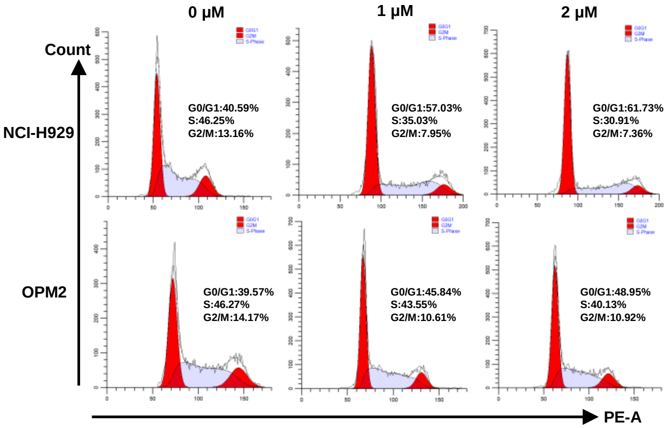

Supplementary Figure 2

A

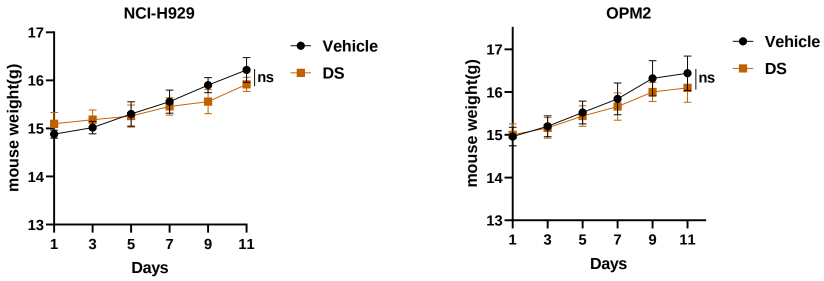

Supplementary Figure 3

A

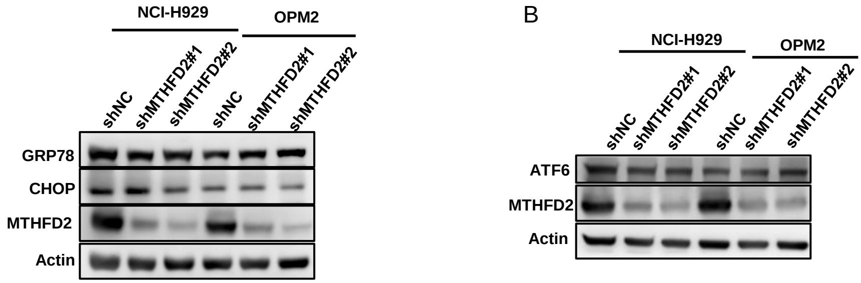

Supplementary Figure 4

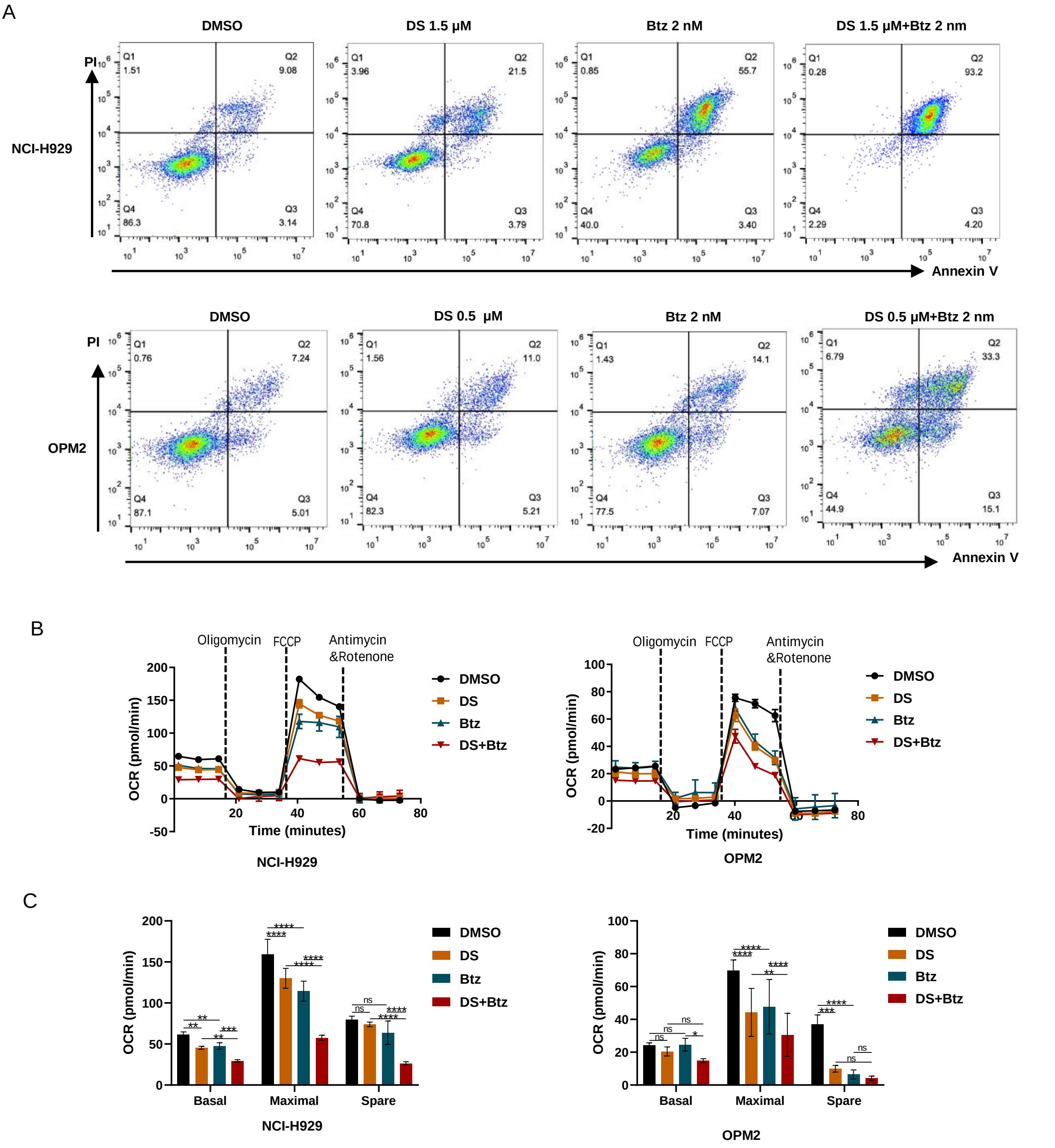

Supplementary Figure 4

D

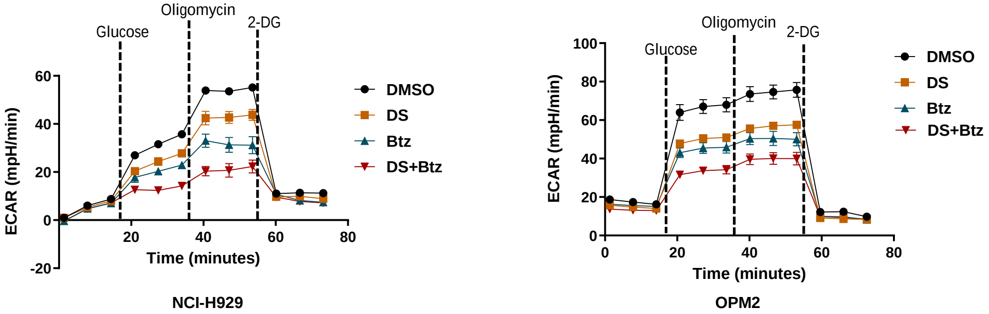

E

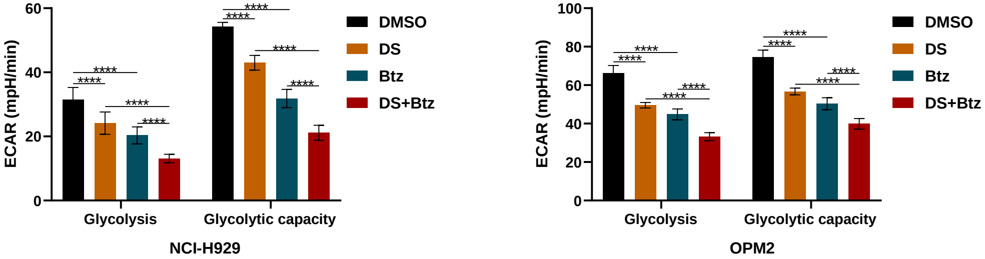

F

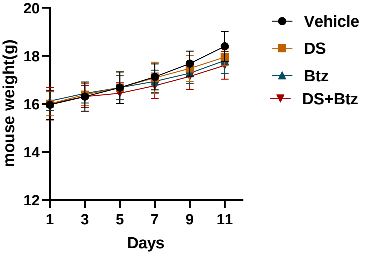

G

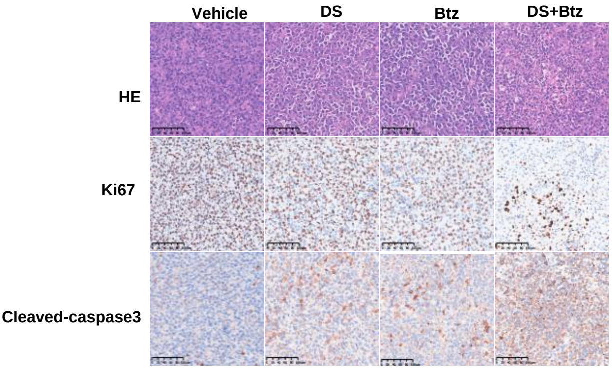

Supplement: Supplementary file 2 — supplementary figures [file 41420_2025_2498_MOESM2_ESM.pdf]
