# Supplementary material for: Targeting MTHFD2 alters metabolic homeostasis and synergizes with bortezomib to inhibit multiple myeloma
Source: Cell Death Discov. 2025 Apr 25;11:201. doi: 10.1038/s41420-025-02498-6 (PMC12032361; doi:10.1038/s41420-025-02498-6)

Figure.1

G

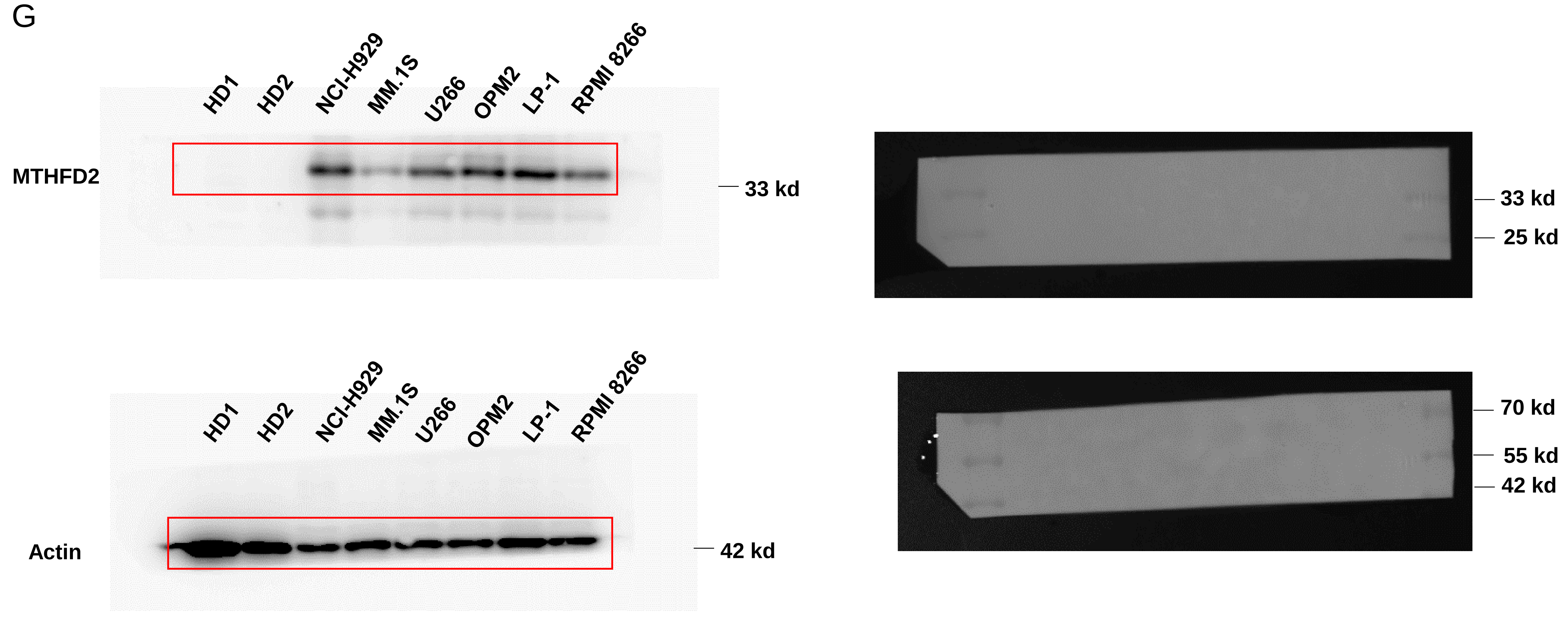

HD1
HD2
NCI-H929
MM.1S
U266
OPM2
LP-1
RPMI 8266
MTHFD2
33 kd
HD1
HD2
NCI-H929
MM.1S
U266
OPM2
LP-1
RPMI 8266
Actin
42 kd
33 kd
25 kd
70 kd
55 kd
42 kd

Figure.2

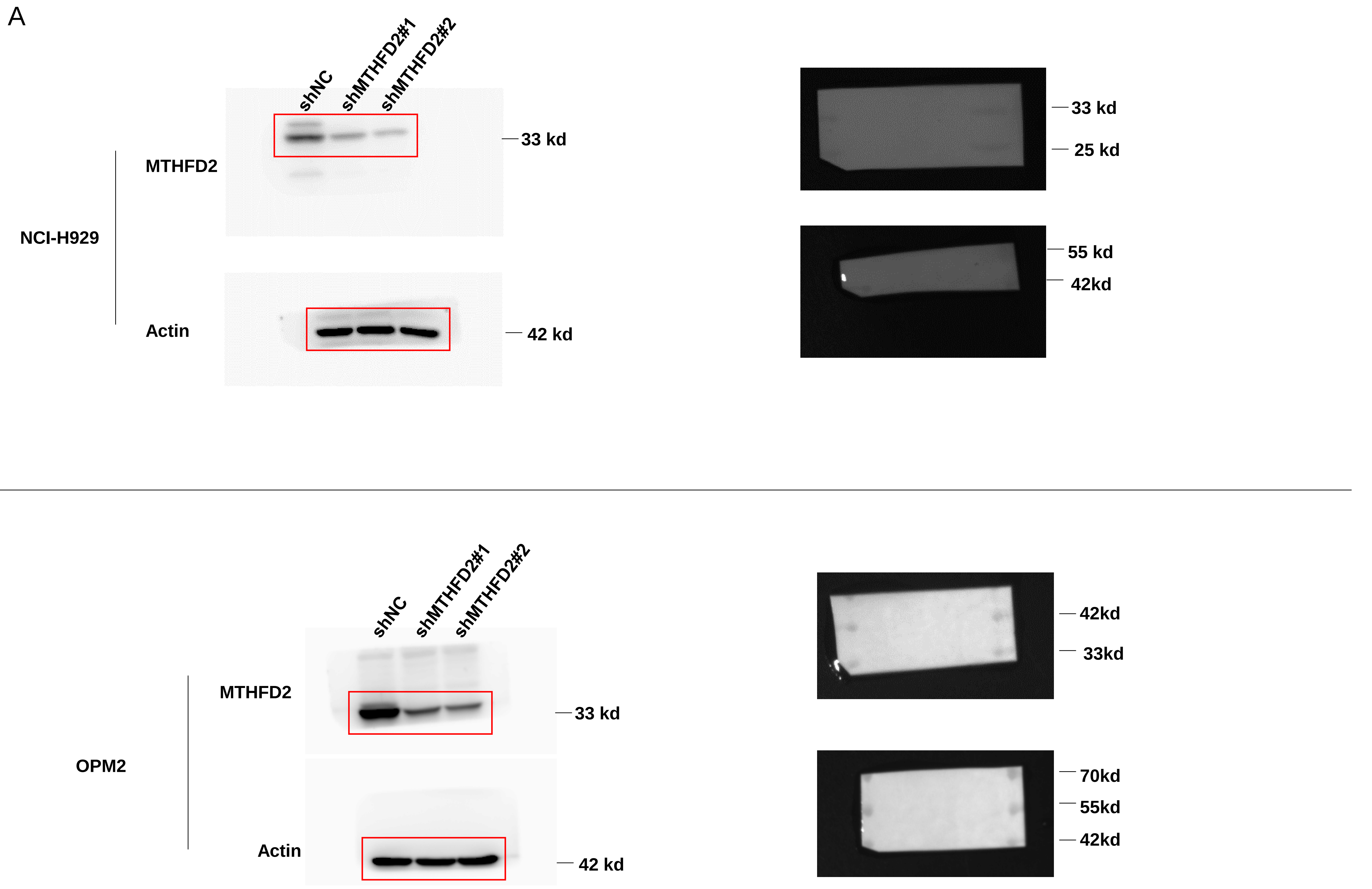
A
shNC
shMTHFD2#1
shMTHFD2#2
MTHFD2
NCI-H929
33 kd
Actin
42 kd
33 kd
25 kd
55 kd
42kd
shNC
shMTHFD2#1
shMTHFD2#2
MTHFD2
OPM2
33 kd
Actin
42 kd
42kd
33kd
70kd
55kd
42kd

Figure.2

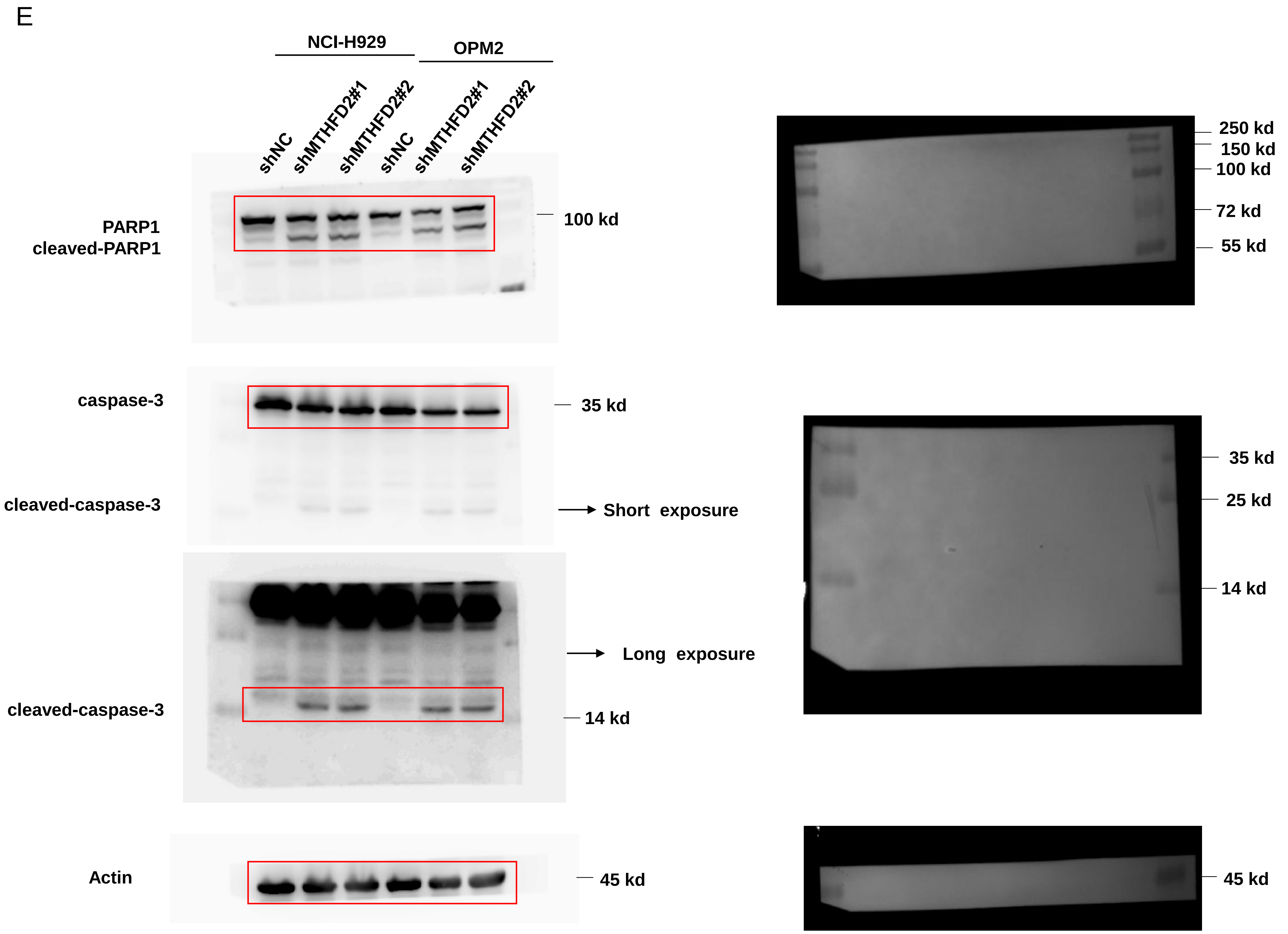

E
NCI-H929
OPM2
shNC
shMTHFD2#1
shMTHFD2#2
shNC
shMTHFD2#1
shMTHFD2#2
PARP1
cleaved-PARP1
100 kd
250 kd
150 kd
100 kd
72 kd
55 kd
caspase-3
35 kd
cleaved-caspase-3
Short exposure
35 kd
25 kd
14 kd
Long exposure
cleaved-caspase-3
14 kd
Actin
45 kd
45 kd

Figure.2

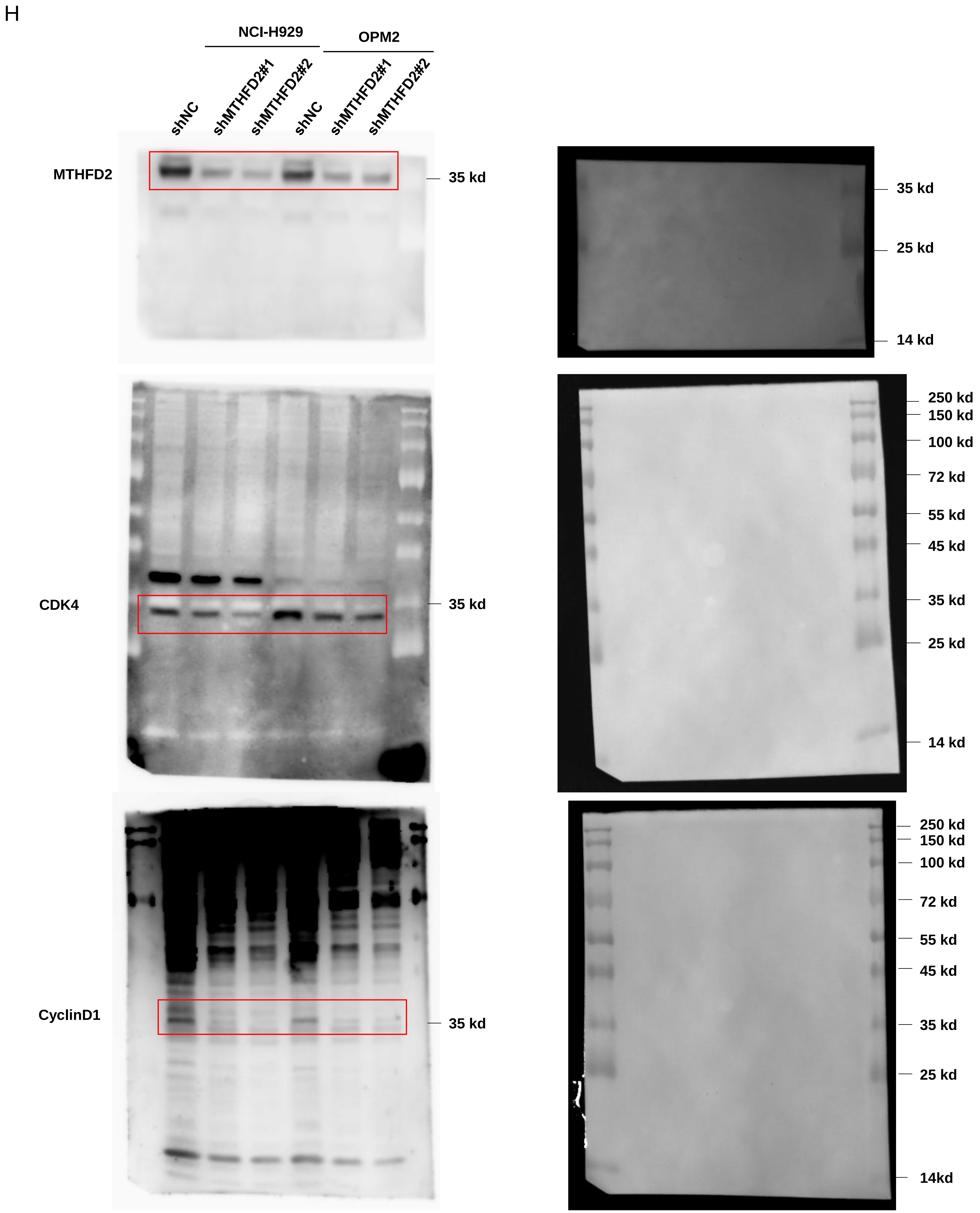
H
NCI-H929
OPM2
shNC
shMTHFD2#1
shMTHFD2#2
shNC
shMTHFD2#1
shMTHFD2#2
MTHFD2
35 kd
35 kd
25 kd
14 kd
CDK4
35 kd
250 kd
150 kd
100 kd
72 kd
55 kd
45 kd
35 kd
25 kd
14 kd
CyclinD1
35 kd
250 kd
150 kd
100 kd
72 kd
55 kd
45 kd
35 kd
25 kd
14kd

Figure.2

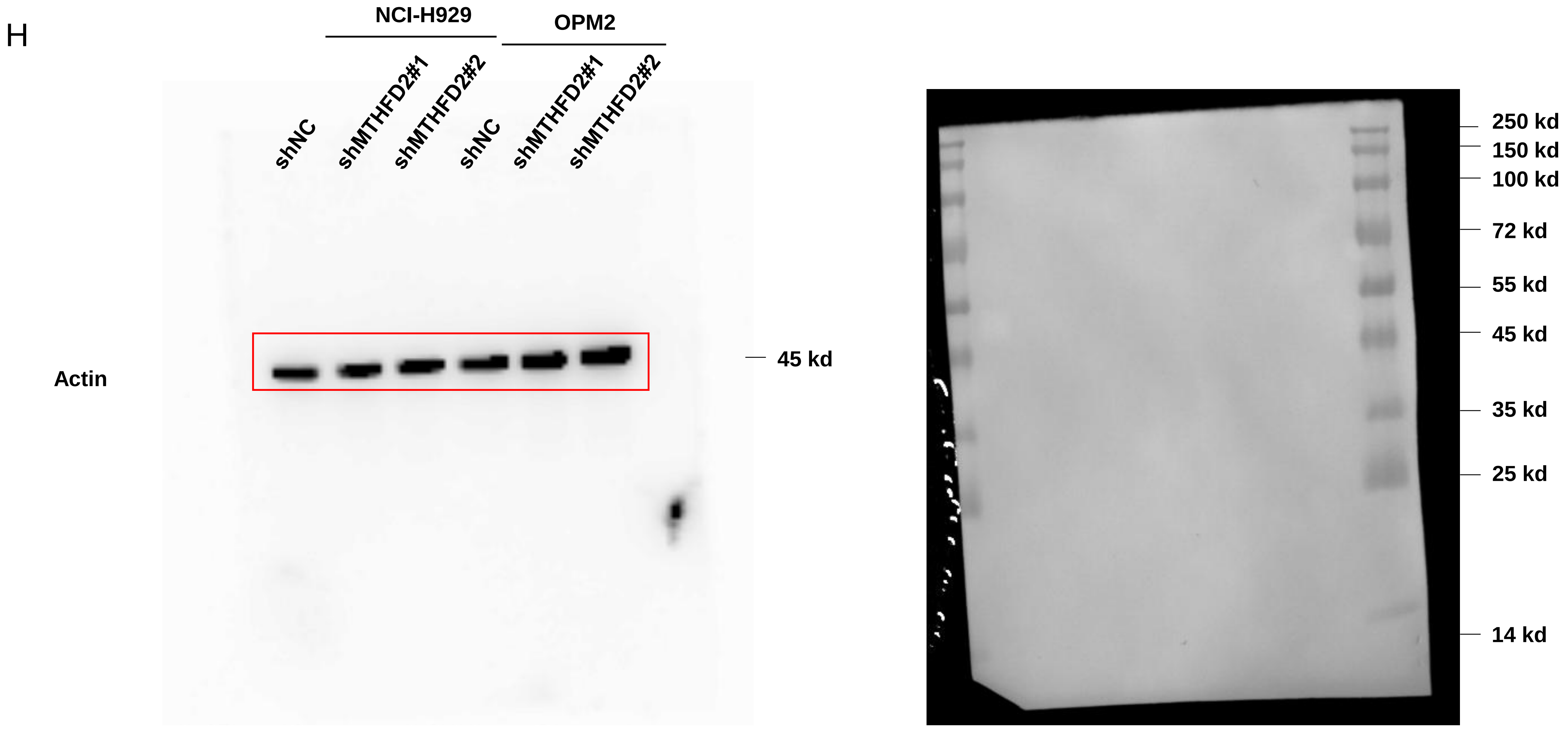

Figure.3

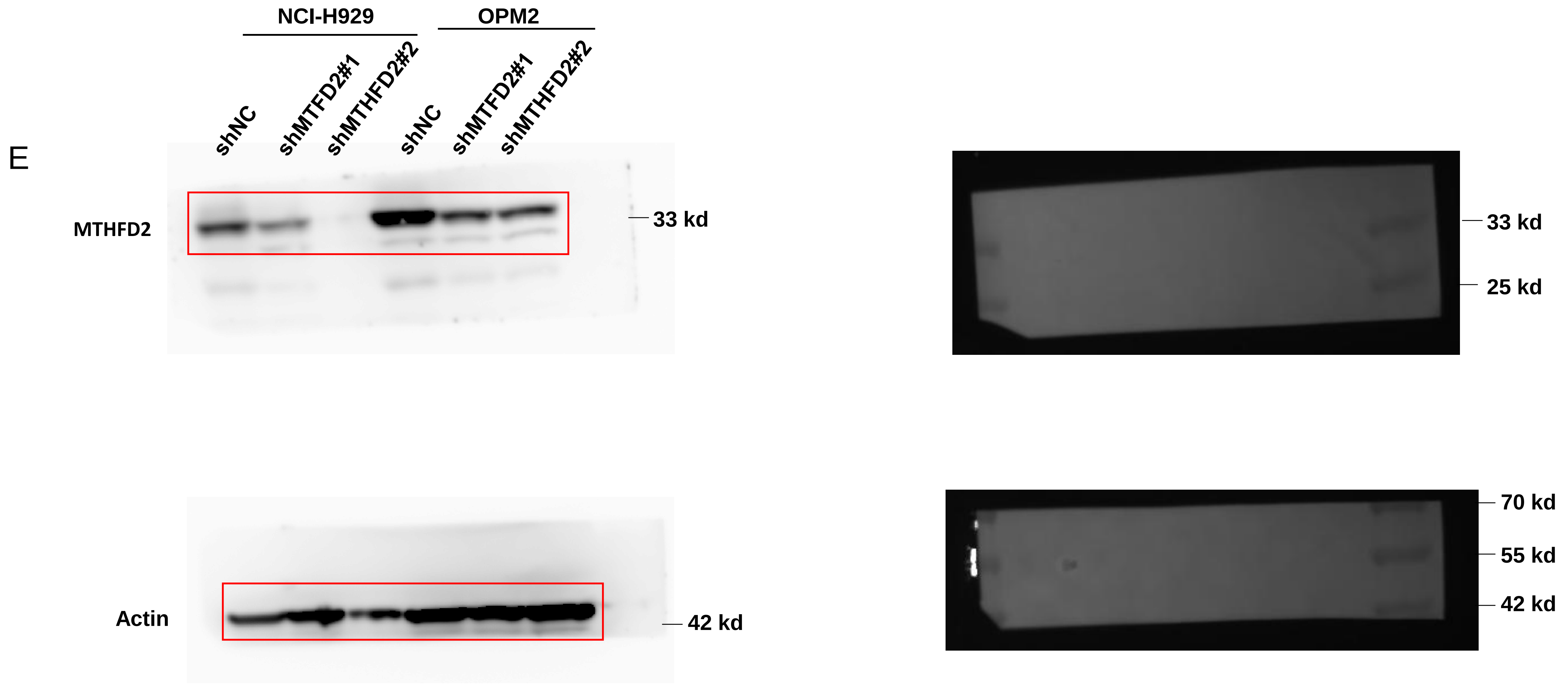

Figure.4

F

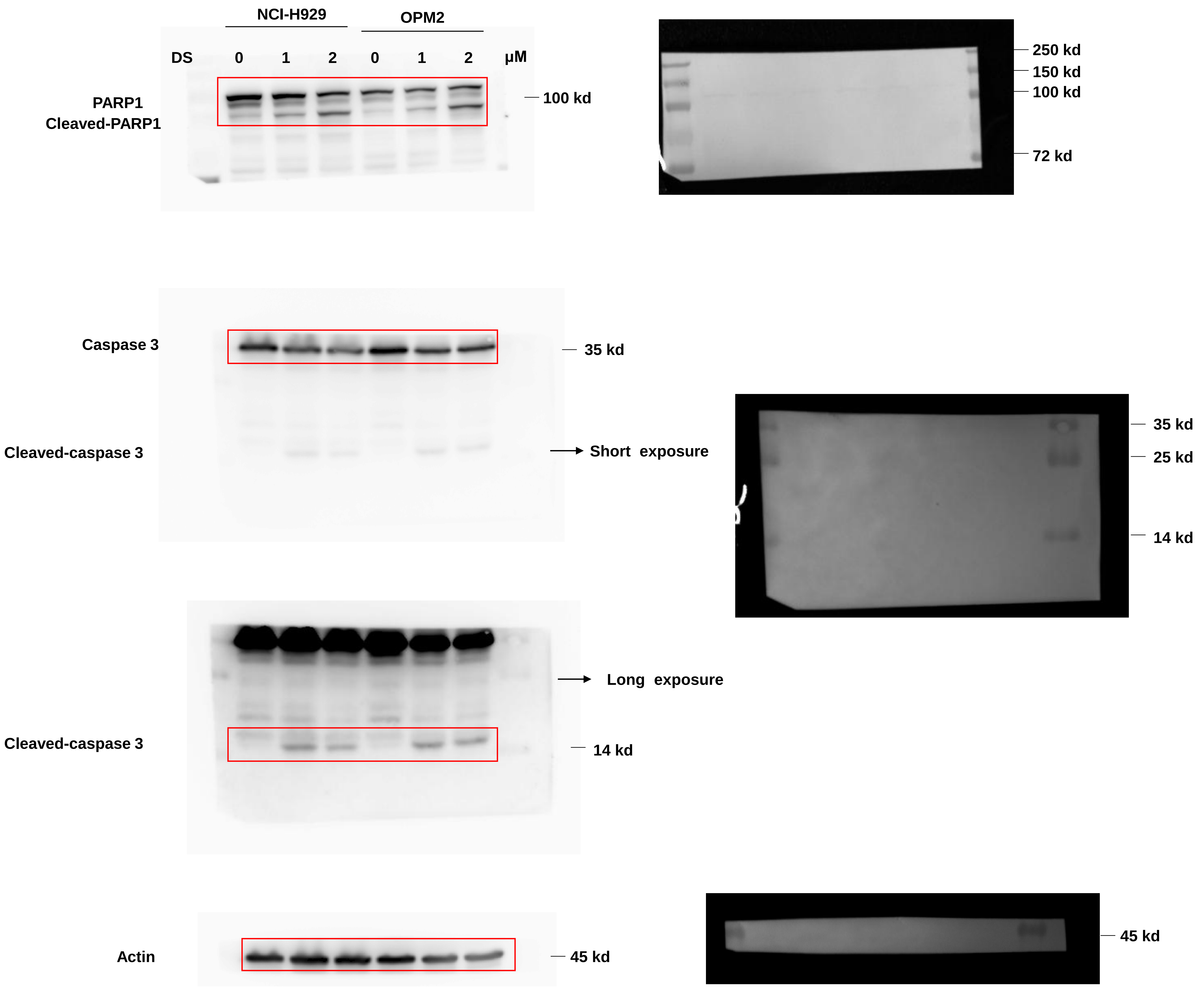

NCI-H929
OPM2
DS
0
1
2
0
1
2
μM
PARP1
Cleaved-PARP1
100 kd
250 kd
150 kd
100 kd
72 kd
Caspase 3
35 kd
Cleaved-caspase 3
Short exposure
35 kd
25 kd
14 kd
Long exposure
Cleaved-caspase 3
14 kd
Actin
45 kd
45 kd

Figure.4

H

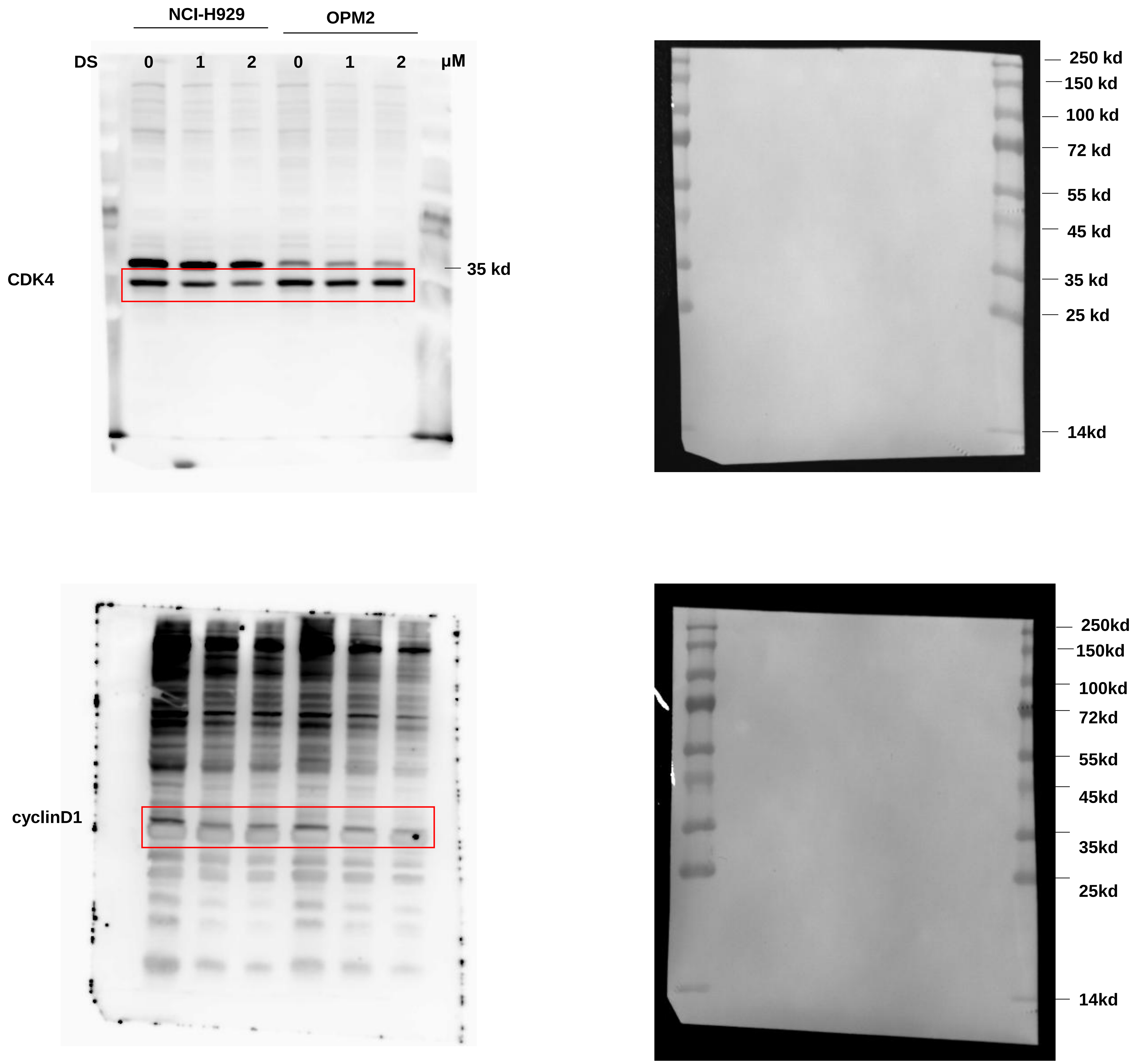

NCI-H929
OPM2
DS
0
1
2
0
1
2
μM
CDK4
35 kd
250 kd
150 kd
100 kd
72 kd
55 kd
45 kd
35 kd
25 kd
14kd
cyclinD1
250kd
150kd
100kd
72kd
55kd
45kd
35kd
25kd
14kd

Figure.4

H

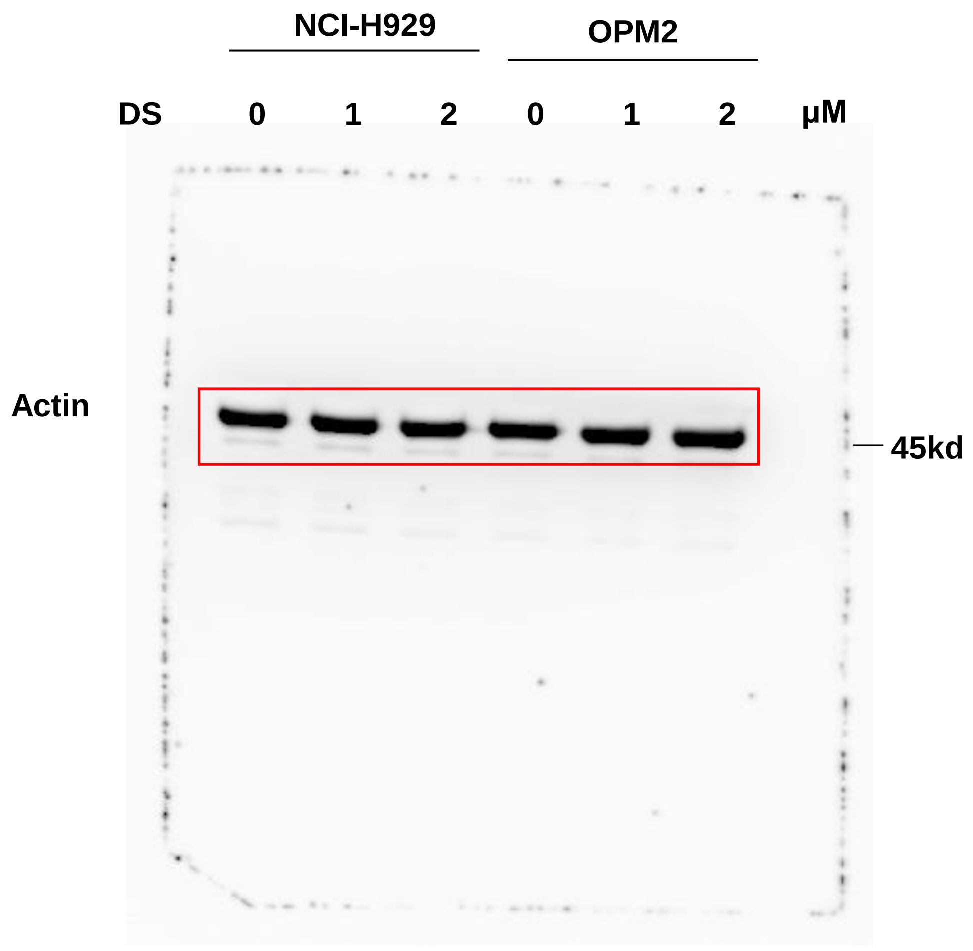
NCI-H929
OPM2
DS 0 1 2 0 1 2 μM
Actin
45kd

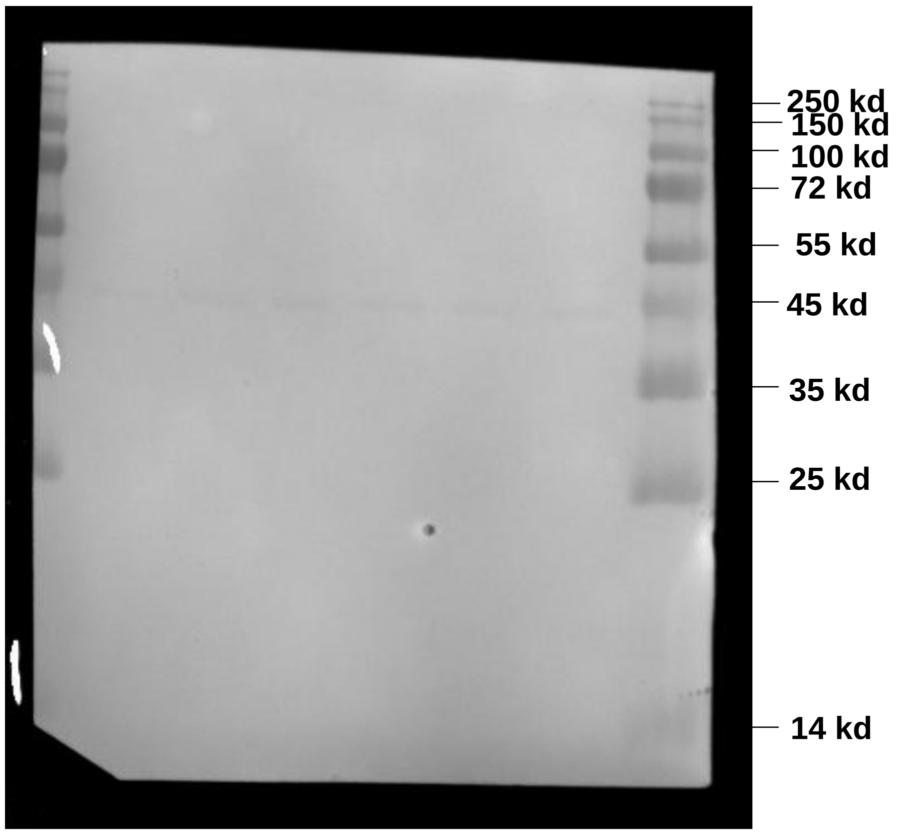
250 kd
150 kd
100 kd
72 kd
55 kd
45 kd
35 kd
25 kd
14 kd

Figure.7

D

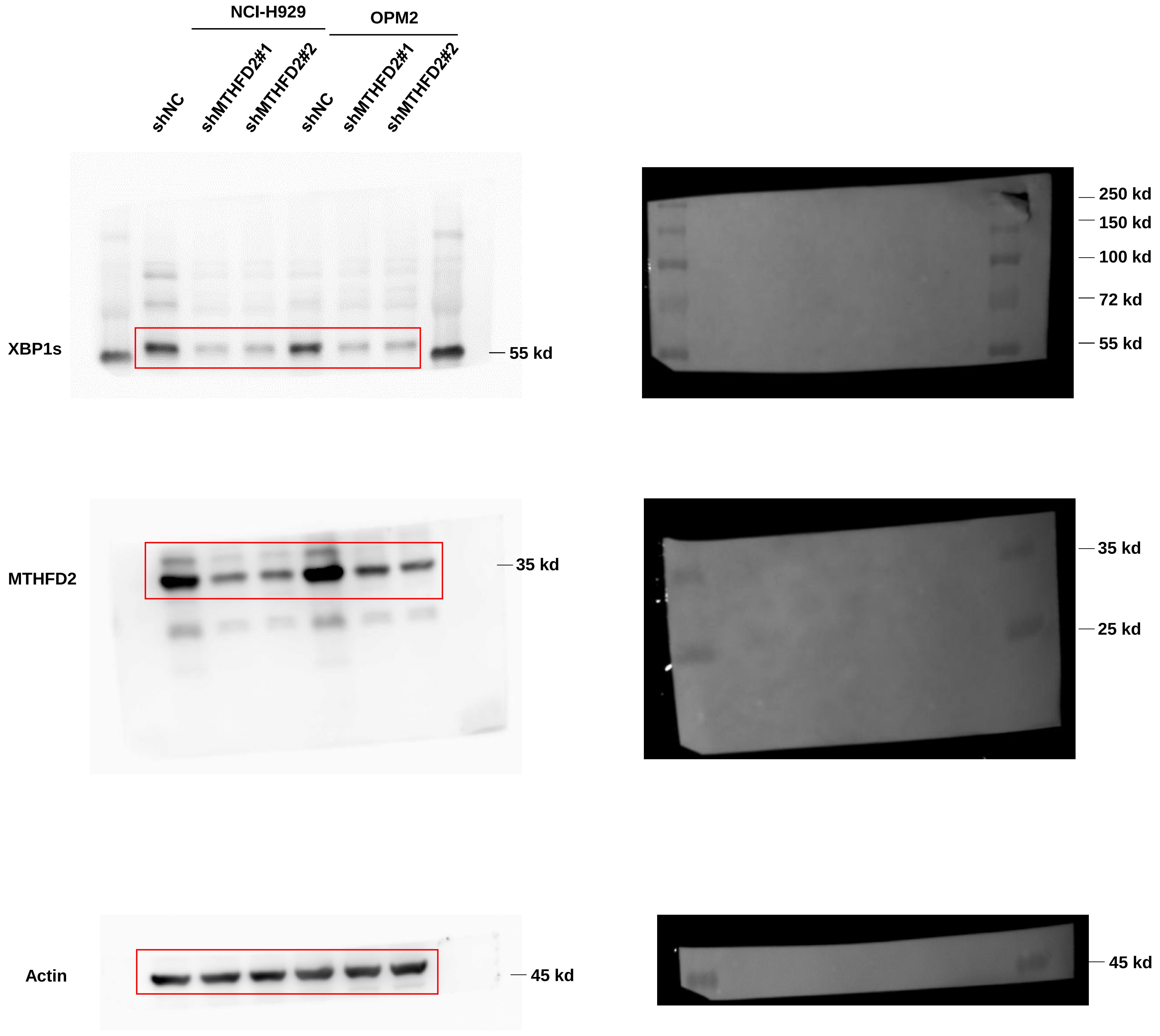

NCI-H929
OPM2
shNC
shMTHFD2#1
shMTHFD2#2
shNC
shMTHFD2#1
shMTHFD2#2
XBP1s
55 kd
250 kd
150 kd
100 kd
72 kd
55 kd
MTHFD2
35 kd
35 kd
25 kd
Actin
45 kd
45 kd

Figure.7

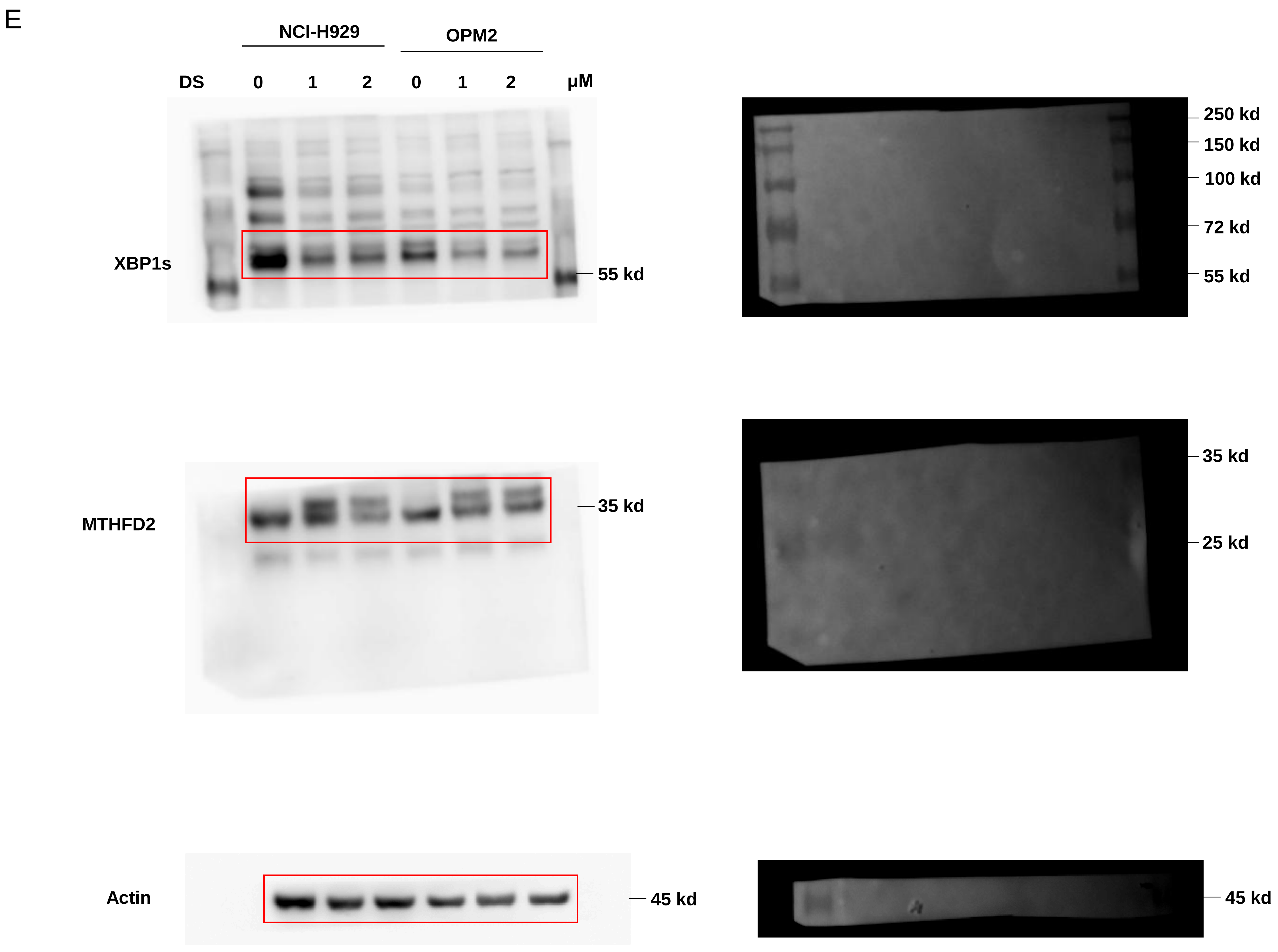
E
NCI-H929
OPM2
DS
0
1
2
0
1
2
μM
XBP1s
55 kd
250 kd
150 kd
100 kd
72 kd
55 kd
MTHFD2
35 kd
35 kd
25 kd
Actin
45 kd
45 kd

Figure.7

F

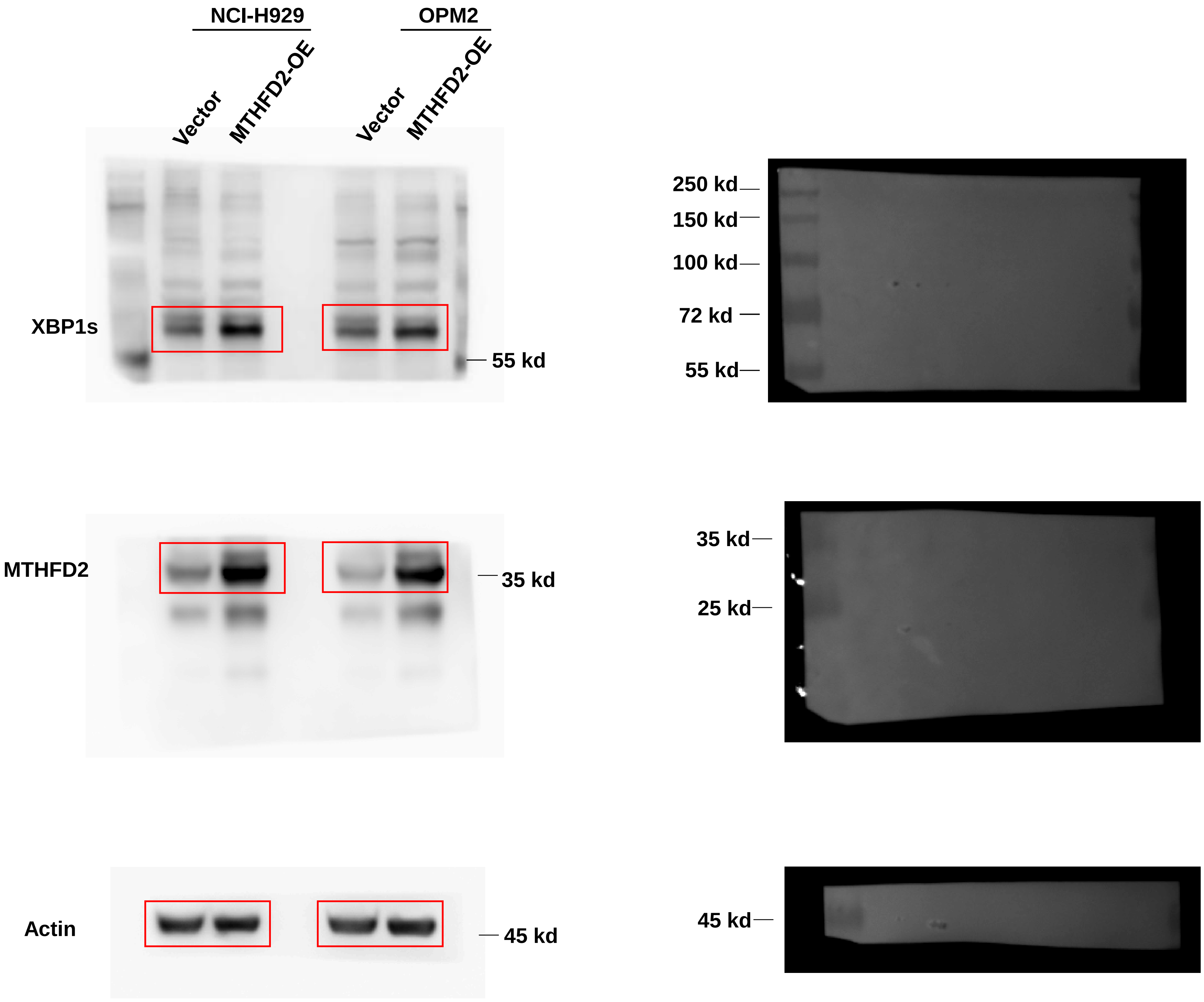
NCI-H929
OPM2
Vector
MTHFD2-OE
Vector
MTHFD2-OE
XBP1s
55 kd
MTHFD2
35 kd
Actin
45 kd
250 kd
150 kd
100 kd
72 kd
55 kd
35 kd
25 kd
45 kd

Figure.7

G

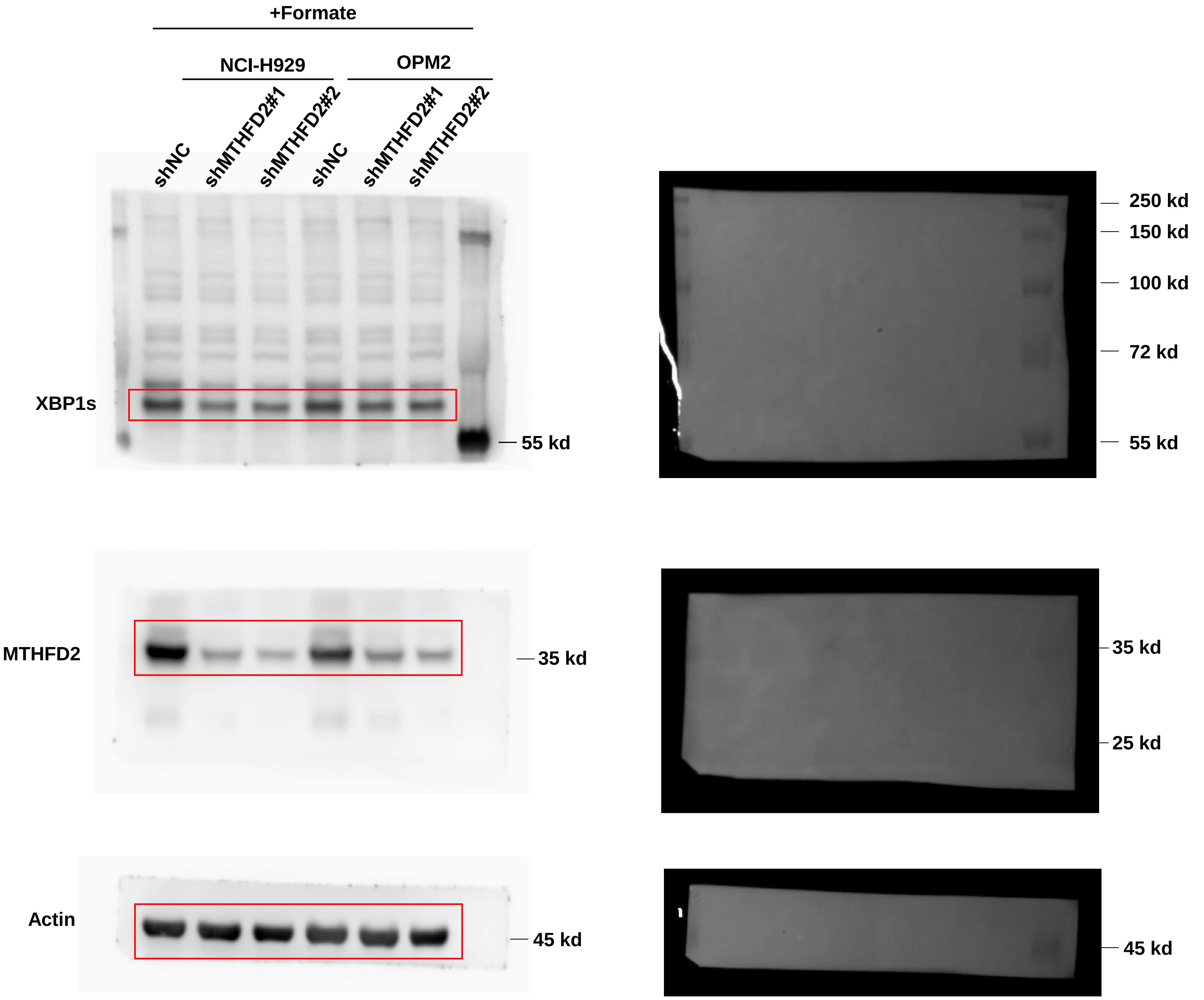

+Formate
NCI-H929
OPM2
shNC
shMTHFD2#1
shMTHFD2#2
shNC
shMTHFD2#1
shMTHFD2#2
XBP1s
55 kd
250 kd
150 kd
100 kd
72 kd
55 kd
MTHFD2
35 kd
35 kd
25 kd
Actin
45 kd
45 kd

Figure.7

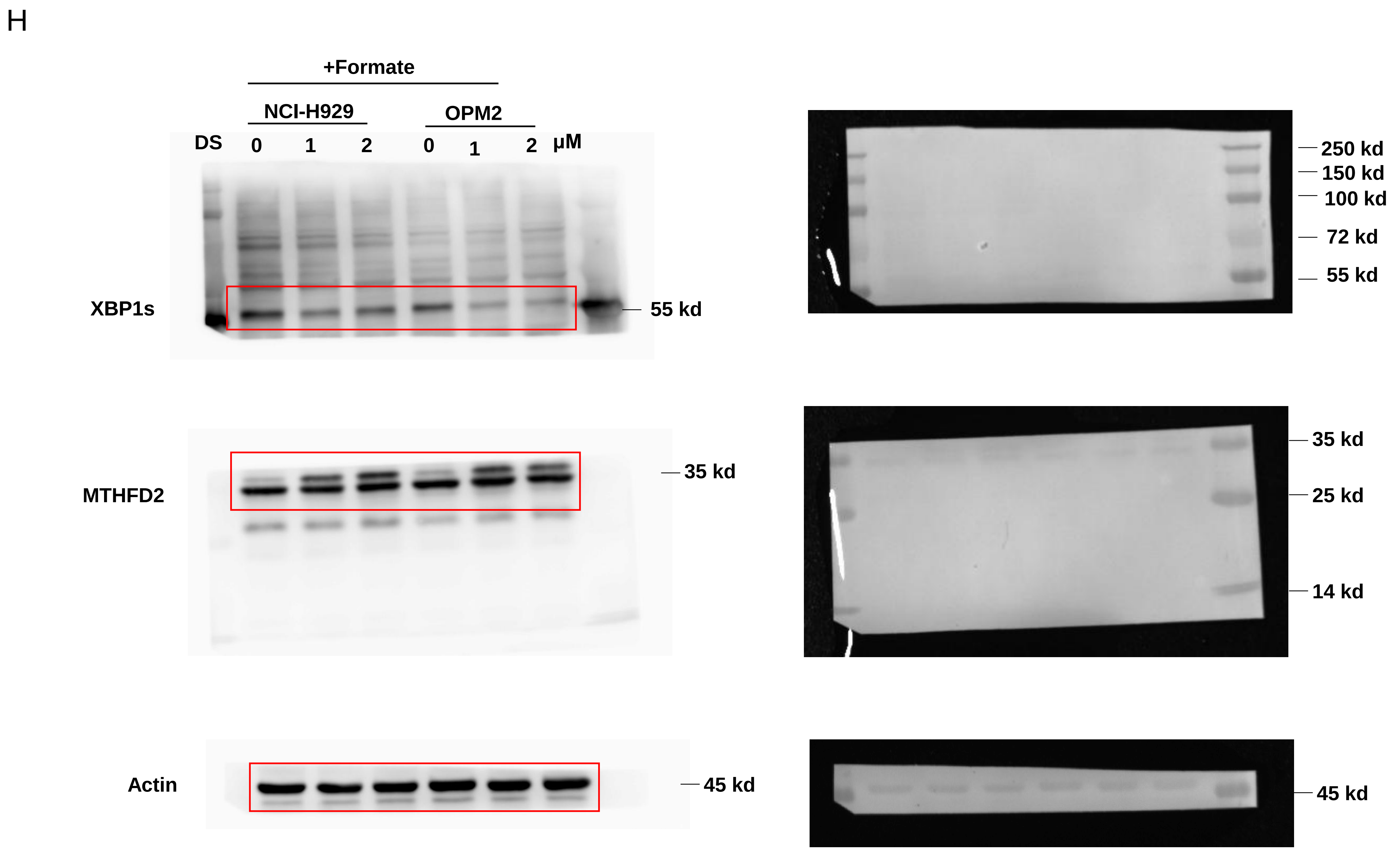
H
+Formate
NCI-H929
OPM2
DS
0
1
2
0
1
2
μM
XBP1s
55 kd
250 kd
150 kd
100 kd
72 kd
55 kd
MTHFD2
35 kd
35 kd
25 kd
14 kd
Actin
45 kd
45 kd

Supplementary Figure 3

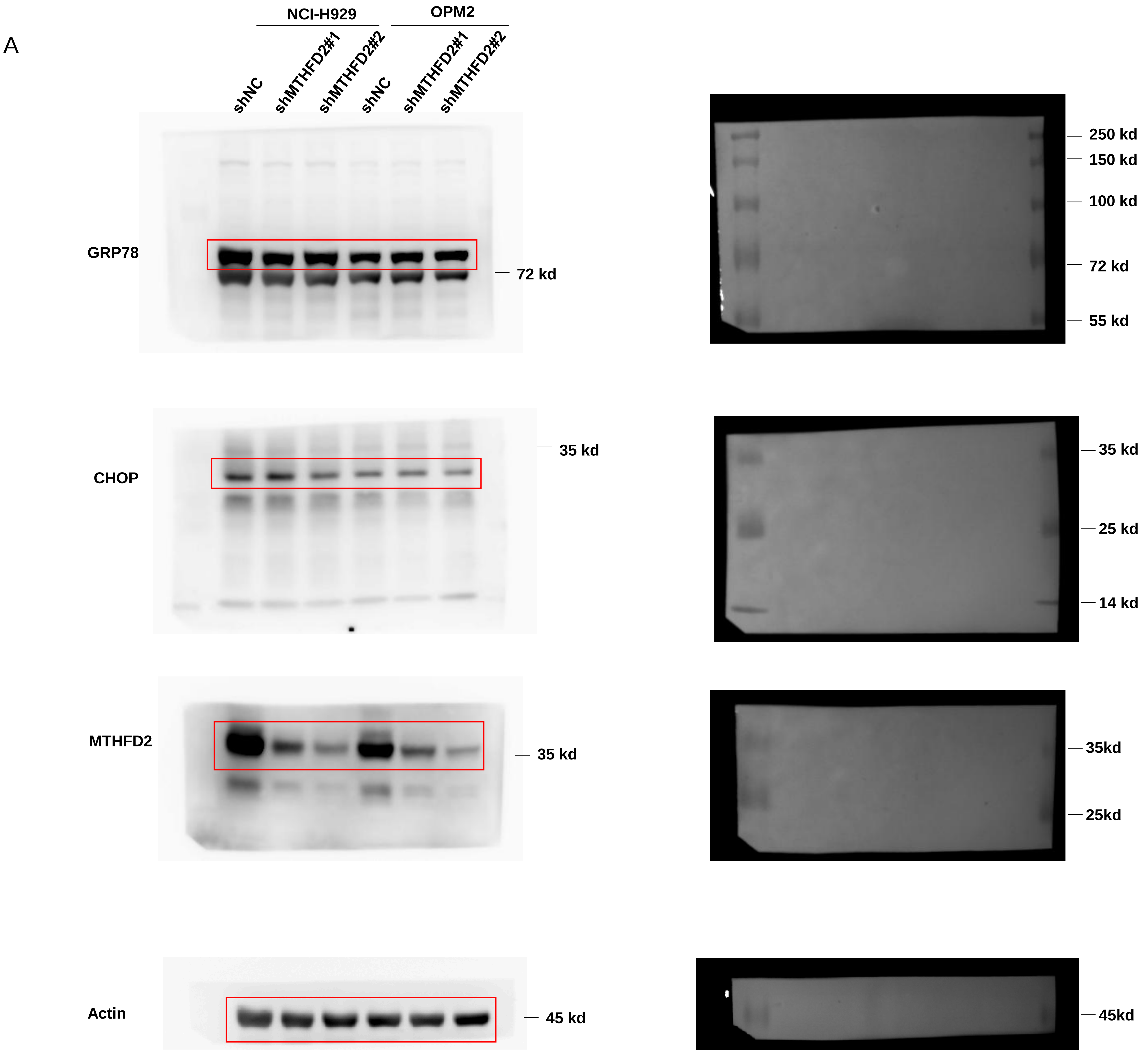

Supplementary Figure 3

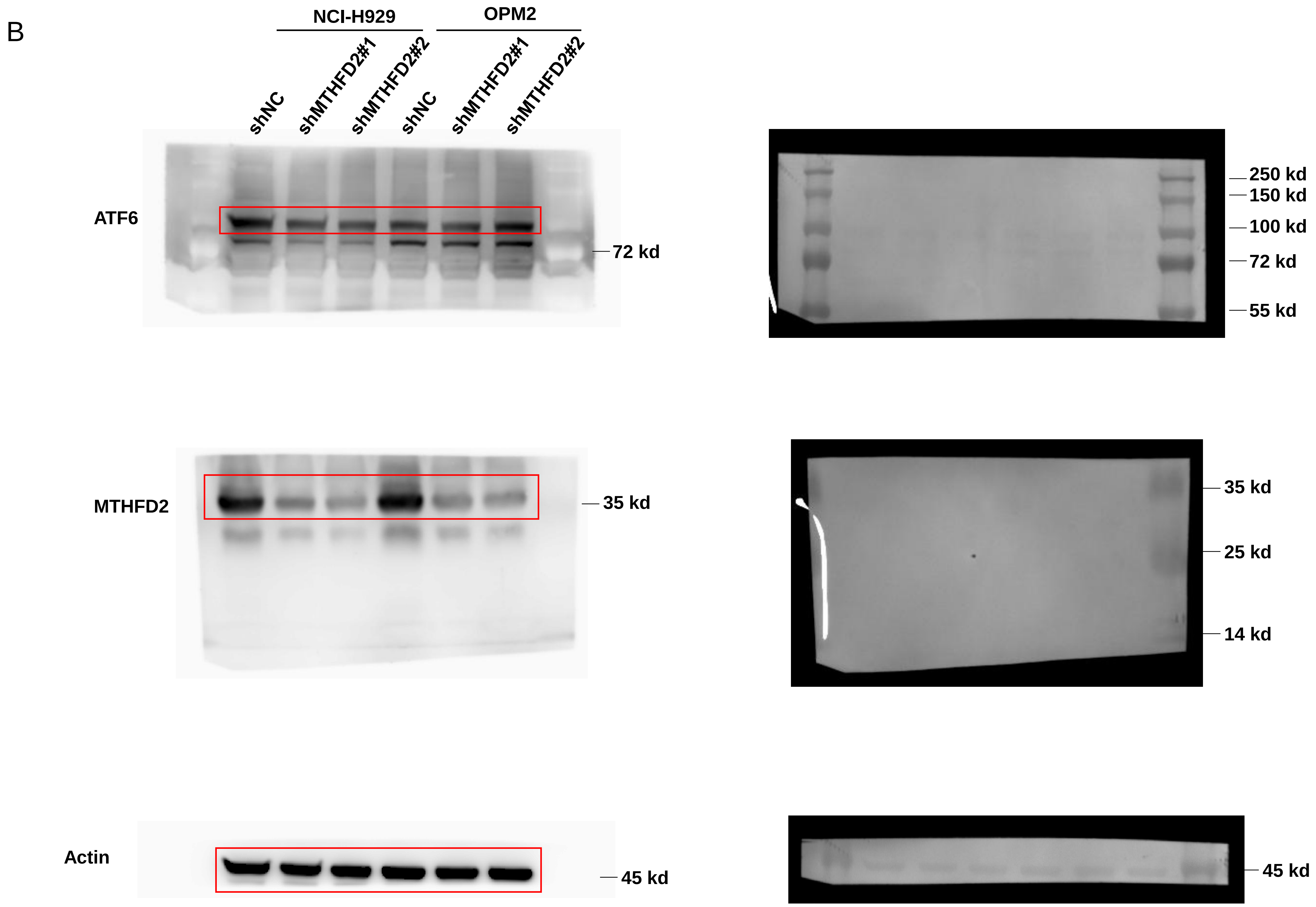

Supplement: Supplementary file 3 — original western blots [file 41420_2025_2498_MOESM3_ESM.pdf]
